# Diurnal temperature range and hospital admission due to cardiovascular diseases: A systematic review and meta-analysis study^☆^

**DOI:** 10.1016/j.ijcrp.2025.200487

**Published:** 2025-08-15

**Authors:** Hamidreza Aghababaeian, Mostafa Hadei, Mahsa Sepasian, Masoumeh Gharaee, Ladan Araghi Ahvazi, Rahim Sharafkhani, Mohammad Zarei

**Affiliations:** a Department of Health in Emergencies and Disasters, Dezful University of Medical Sciences, Dezful, Iran; b Center for Climate Chang and Health Research (CCCHR), Dezful University of Medical Sciences, Dezful, Iran; c Universal Scientific Education and Research Network (USERN), Dezful University of Medical Sciences, Dezful, Iran; d Department of Health in Emergencies and Disasters, Tehran University of Medical Sciences, Tehran, Iran; e Climate Change and Health Research Center (CCHRC), Institute for Environmental Research (IER), Tehran University of Medical Sciences, Tehran, Iran; f Department of Climatology, Faculty of Geographical Sciences, Kharazmi University, Tehran, Iran; g School of Public Health, Khoy University of Medical Sciences, Khoy, Iran; h Department of Nursing, Shirvan School of Nursing, North Khorasan University of Medical Sciences, Bojnurd, Iran

**Keywords:** Diurnal temperature range, Climate change, Disaster, Cardiovascular, Health

## Abstract

This study investigated the impact of the diurnal temperature range (DTR) on hospitalizations related to cardiovascular disease (CVD). Following the PRISMA protocol, a systematic review and meta-analysis searched various databases for English studies using keywords related to DTR and CVD up to June 1, 2023. A random-effects meta-analysis model was utilized to aggregate previous estimates of DTR effects on CVD admissions. The findings revealed that a 1 °C increase in DTR is associated with a 1.5 % increase in all CVD hospitalizations (95 % CI: 0.2 %, 3 %). Additionally, for each 1 °C increase in DTR, admissions due to acute myocardial infarction (AMI) and heart failure increased by relative risks (RR) of 1.02 (95 % CI: 1.01, 1.03) and 1.04 (95 % CI: 1.03, 1.04), respectively. Our analysis showed that a 1 °C increase in DTR was associated with a 0.9 % increase in all CVD hospital admissions among those aged ≥65 years (95 % CI: 0.3 %, 1.6 %). The overall estimates indicated that DTR (per 1 °C increment) was associated with a 0.6 % (95 % CI: 0.2 %, 1.1 %) increase in females and a 1.7 % (95 % CI: 1.3 %, 2.2 %) increase in males. It was statistically significant for elderly individuals, corresponding to a 4.5 % increase in stroke risk (RR: 1.045 [95 % CI: 1.01, 1.07]). Overall, this study emphasizes that daily fluctuations in DTR increase the hospitalization risk in cardiovascular patients, highlighting the need to consider the effects of DTR on cardiovascular health, especially among vulnerable age and sex groups.

## Highlights

• First systematic review on Diurnal Temperature Range and cardiovascular admissions.
• A 1 °C rise in Diurnal Temperature Range increases cardiovascular admissions by 1.5 %.
• A 1 °C rise in Diurnal Temperature Range increases elderly stroke admissions risk by 4.5 %.

## 1 Introduction

The effect of temperature on human health has been well established, and numerous studies have been conducted to investigate the impacts of temperature [[1], [2], [3], [4], [5]]. Furthermore, with the increase in global climate change, researchers have sought to study its consequences, especially temperature changes [[6], [7], [8], [9]]. Several studies have examined the effects of various temperature indices, such as average, minimum, and maximum temperature, on health [3,[8], [9], [10], [11], [12]]. Other studies have chosen another index of temperature, the index of daily temperature changes (DTR), in relation to human health [2,4,7,[13], [14], [15], [16]]. One of the most important indicators is DTR, which is a meteorological index indicating the degree of weather stability related to climate change and urbanization [17]. The DTR is defined as the difference between the maximum and minimum temperatures in a single day [7], and it is a significant indicator of climate change because of the more detailed information it provides compared to the average temperature [18,19]. Given that, during the 20th century, minimum temperatures increased three times faster than maximum temperatures, DTR is currently declining in most parts of the world. However, although the DTR is decreasing in the context of long-term climate change, the short-term effects of high DTR on human health are persistent in many countries and regions [20]. Therefore, researchers have used this index to recognize the negative effects of temperature on human health, especially on cardiovascular and respiratory diseases [4,21].

High variations in DTR cause double environmental stress and adverse effects on human health, especially for those suffering from chronic conditions, such as cardiovascular and respiratory diseases [2,4,22]. This is because it leads to a decrease in the ability to adapt to wide temperature changes throughout the day [20]. Researchers have shown that a high DTR can increase blood pressure, heart rate, and oxygen intake [23], increase cardiovascular workload and contribute to cardiac events and even death [19]. Elderly individuals, who are typically more vulnerable to health risks related to heat or cold, especially those who are socially or economically disadvantaged, are a high-risk group that should be considered and monitored. To reduce the adverse effects of DTR on these people, measures such as access to air conditioning or home heating should be provided [24]. Cardiovascular diseases are a leading cause of mortality worldwide and are one of the primary causes of hospitalization [17], making them a significant concern for public health authorities and prompting researchers to pay more attention to this disease category. Consequently, studies on DTR and its effects have been conducted in various regions, including China [25], Hong Kong [26], Korea [27], Taiwan [28], Japan [29], and the United States [30]. Most existing studies have reported that DTR is an independent risk factor for mortality and is directly related to the occurrence of death, hospitalization, and emergency events [10,15,23]. It should be noted that the majority of these studies have focused on the effects of DTR on CVD mortality, while other studies have been based on data from emergency rooms and hospitalizations [4,13,14,31].

As mentioned, according to previous research, DTR is one of the most important and influential health indicators, and cardiovascular diseases are among the most sensitive to temperature changes. Therefore, the purpose of this research was to determine the effects of daily temperature changes on the risk of hospitalization. Based on these findings, the results of the present study and detailed examination of the effects of daily temperature changes, in addition to a comprehensive and general view of the impacts of this index on cardiovascular patients, can be used for prevention or planning to adapt to adverse weather conditions.

## 2 Methods

This systematic review and meta-analysis was conducted following the recommendations of the PRISMA guidelines, with no time limits on the search until the beginning of June 2023. The authors searched for all scientific articles related to the effects of daily temperature changes on the rate of cardiovascular hospitalizations worldwide recorded in English in the international databases Scopus, EMBASE, PubMed, and Web of Science. Reliable scientific websites and sources were also searched to find gray literature, and the Google Scholar search engine was utilized to aid in the search for relevant articles. Additionally, manual searches and reference checking of the obtained articles were used to increase the validity of the search. The study protocol was preregistered in PROSPERO (CRD42022343851).

The search strategy and selection of keywords were finalized based on a review of relevant previous studies and medical subject headings (MeSH and EMTREE) using the Thesaurus website and consultation with library experts and the research team. Keywords related to diurnal temperature range and cardiovascular problems were used to construct the search syntax for each database.

Diurnal temperature range OR temperature change OR temperature variation AND hospital OR “hospitalization OR emergency room OR emergency department OR outpatient OR morbidity OR health∗ OR disease∗ OR Admission∗ OR “Adverse effect” OR affect∗ AND cardiovascular OR cardiac OR coronary artery diseases OR stroke” OR “myocardial infarction” OR “coronary revascularization” OR “angina pectoris OR myocardial infarct∗ OR coronary event” OR ‘‘heart attack’’, ‘‘Q wave OR infarct∗’’OR ‘‘non-Q wave infarct∗’’OR ‘‘Acute coronary syndrome’ ’OR ‘‘QWMI’’OR ‘‘NQWMI’’OR‘STEMI’’OR ‘‘NSTEMI’’OR ‘‘coronary infarct∗’’OR ‘‘heart infarct∗’’OR ‘‘myocardial thrombosis’’, OR‘ ‘coronary thrombosis’ ’OR’’congestive heart failure’’ OR ‘‘heart failure’’

Predetermined decision rules were utilized to screen the identified studies. After removing duplicate articles, two reviewers (H.A. and M.S.) independently evaluated the titles and abstracts of all the articles found through the literature search, with an additional investigator (M.H.) randomly reviewing 10 % of the studies. The investigators (H.A. and L.AA.) then reviewed the full texts of the potentially eligible papers, resolving any inconsistencies through mutual agreement or by consulting another reviewer (M.S.). In cases of disagreement in grading, a definitive grade was adopted after consensus and agreement were reached. Consensus was reached in all patients. The following data were extracted from the articles.

• Article number in this study
• Study reference
• Year of the study
• First author of the study
• Study type
• Country under study
• Health indices
• Study results (adverse health effects)

### 2.1 - Inclusion criteria

• Studies that used real data and evaluated CVD incidence based on the International Classification of Diseases (ICD) were included.
• Studies that reported a quantitative effect estimate appropriate for the analysis.
• For example, risk estimates (RE), incidence rate ratio (IRR), odds ratio (OR), relative risk (RR), regression coefficient, and percent change in morbidity
• The main variables of that study were daily temperature changes and cardiovascular problems.
• Studies that investigated the effects of the temperature of the external environment.

### 2.2 Exclusion criteria

• Articles published in languages other than English.
• The full texts of the articles were not available.
• This article examines the consequences of daily temperature changes on plants and animals.
• The results of these studies were generated via computer simulation or laboratory work. Studies that investigated the effects of the temperature of the internal environment.

### 2.3 Critical appraisal of study quality

In this study, we utilized the risk of bias (RoB) assessment tool to evaluate the quality of each study, modified according to the OHAT method [32]. This appraisal was conducted in three stages: first, the RoB assessment in each study; second, the assessment of the quality of evidence across studies; and third, the evaluation of the strength of evidence. Each stage of appraisal was independently performed by two researchers (HA and MS). The results for each component were discussed to reach an agreement, and in cases of disagreement, resolution was achieved through consultation with RSH and MH. The key criteria included exposure assessment, outcome assessment, and confounding bias. The overall risk of bias was determined based on the combination of these three components. For instance, if all three components were rated as "High" (H), the overall risk was assessed as "High". Various combinations, such as "probably high" (PH) and "probably low" (PL), have also been meticulously examined [[32], [33], [34]].

## 3 Meta-analysis

The data extracted from the article included author, year of publication, study location (country and city or city), study period, sample size, sex, age group, statistical model, average (±standard deviation) observed DTR, risk measure (RR, OR, and percent change), risk estimates with 95 % confidence intervals, and model adjustments.

Since the included studies evaluated multiple cause-specific cardiovascular outcomes in various sex and age groups, we investigated whether a meta-analysis could be conducted for each sex-age-outcome combination. In this regard, if the number of relevant studies for each sex-age-outcome combination was equal to or greater than three, a meta-analysis was performed. Therefore, twelve sets of meta-analyses were conducted: 1) for both sexes and all age groups and all cardiovascular causes; 2) for both sexes and all age groups and for both sexes and all age groups and stroke; 4) for both sexes and all age groups and acute myocardial infarction (AMI); 5) for both sexes and elderly individuals (≥65 years old) and all cardiovascular causes; 6) for both sexes and people aged <65 years old and all cardiovascular causes; 7) for all age groups and all cardiovascular causes; 8) for all age groups and all cardiovascular causes; 9) for both genders and people aged <65 years and stroke; 10) for both genders and elderly individuals (≥65 years old) and stroke; and 11) for all age groups and stroke; and 12) for males and all age groups.

We selected one risk estimate from each study for inclusion in the meta-analysis. Some studies have reported one risk estimate for each sex-age combination. The others reported risk estimates for several lag days. In this case, we followed the approach previously used in meta-analyses of time-series studies [35,36]. To select one risk estimate from each study, the most statistically significant estimate or the largest estimate was chosen. One study that investigated four cities in South Korea did not report overall results [37]. Therefore, we pooled their results for the four cities and then included the overall estimates in the final meta-analysis.

We selected relative risk (RR) as our risk measure of interest. Therefore, other risk measures reported in the studies were converted to RRs. Percent changes were converted to RRs by dividing the percentage by 100 and adding the outcome to 1 (RR = 1 + (percent change/100)).

STATA v.17 (STATA Corp., College Station, TX) was used for the meta-analysis. Before conducting the meta-analysis, the inconsistency index (I^2^) test was performed to investigate the heterogeneity across the studies. The I^2^ statistic determines the percentage of total variation across studies due to heterogeneity rather than chance. This measure is calculated based on the equation provided by Higgins et al. [38] and ranges from 0 to 100 %. In our study, when I^2^ exceeded 50 %, a random effect model was constructed using the Hunter–Schmidt method; otherwise, a fixed effects model was applied. A funnel plot and Egger test were used to evaluate publication bias. A significance level of 0.05 was used in all analyses.

## 4 Results

Initially, 432 articles were identified, of which 22 were included in the final review based on the inclusion and exclusion criteria. Sixteen studies were eligible for quantitative evaluation (Flowchart 1). These 16 studies were conducted in the following countries: China (6 studies) [6,7,14,26,39,40], Korea (4 studies) [37,[41], [42], [43]], Iran (1 study) [4], Thailand (1 study) [44], Mediterranean regions (1 study) [45], Russia (1 study) [46], Israel (1 study) [47], and the USA (1 study) [48].

All the studies sought to determine the effect of DTR on CVDs, and most of them used time series and distributed lag nonlinear models (DLNMs) and generalized additive models (GAMs) to determine the relationships between them (S1-Table 1).

A study conducted by Zhu et al. (2021) in China revealed that a cumulative lag effect associated with a **1°C increase in the daily temperature range (DTR)** leads to increased health risks. Specifically, **the incidence of cardiovascular disease (CVD)** increased **by 1.30 % (95 % CI: 0.99**–**1.62 %) at Lag07**, hypertension (HTN) increased **by 1.73 % (95 % CI: 1.30**–**2.15 %) at Lag07**, ischemic heart disease (IHD) increased **by 1.71 % (95 % CI: 1.05**–**2.38 %) at Lag04**, and stroke increased **by 1.49 % (95 % CI: 0.14**–**2.86 %) at Lag07** [14]. Zhai et al. (2021) reported that the highest cumulative risk for CVD hospital admissions occurred at **Lag 0**–**14**, with a relative risk (RR) of **2.190 (95 % CI: 1.404**–**3.416)** [7]. Wang et al. (2013) analyzed the impact of DTR on CVD risk and reported an RR of **0.62 at Lag 01**, suggesting a complex relationship in which an increased DTR may not uniformly elevate CVD risk [6]. Qiu et al. (2013) assessed emergency heart failure (HF) admissions in China and reported a cumulative RR of **3.76 for each 1°C increase in DTR** [26]. Ponjoan et al. (2021) [45] and Phosri et al. (2020) [44] reported significant increases in CVD hospitalizations associated with extreme DTR in **the Mediterranean region and Thailand**, respectively. Lim et al. (2012) reported a positive association between DTR and CVD admissions in **South Korea**, with significant risks for cardiac failure [37]. Aghababaeian et al. (2023) reported that **extremely low DTRs significantly increased cardiovascular admissions in Dezful, Iran**, while **extremely high DTRs were associated with a decrease in admissions** [4]. Lim et al. (2017) investigated the impact of diurnal temperature changes on the incidence of ischemic stroke in South Korea. The study found significant associations between diurnal temperature changes and stroke occurrences. Specifically, a **1°C increase in temperature was linked to a 2.4 % higher risk of acute stroke in men**, and in individuals over **65 years**, a **1°C change corresponded to a 2.7 % higher risk of acute ischemic stroke (RR 1.027, 95 % CI 1.008**–**1.047)** [43]. Shaposhnikov (2014) in Moscow explored the impact of diurnal temperature range (DTR) on outpatient and emergency room (O&ER) admissions for cardiovascular diseases (CVDs). The study revealed a significant **J-shaped relationship between DTR and brain stroke (BS)**, with the lowest risk at **DTR = 4°C**. A **10°C increase in DTR from 4°C to 14°C** was linked to a **26 % rise in brain stroke admissions**, peaking at a **5-day lag** [46]. Vereda et al. (2020) in Israel examined the relationship between diurnal temperature range (DTR) during summer and the risk of stroke/transient ischemic attack (TIA) in individuals aged ≥50 years in Israel. Their findings revealed that a **larger DTR prior to stroke/TIA occurrence was associated with a decreased risk**, with an odds ratio (OR) of **0.96 (95 % CI: 0.95**–**0.97) for a six-day lag** [47]. Lin et al. (2020) reported a significant association between high diurnal temperature range (DTR) and first-ever strokes in China. The risk of stroke increased with rising DTR during both summer and winter. Specifically, **3.65 % (95 % empirical CI 1.81 % to 5.53 %) of first-ever strokes in summer were attributable to high DTR (≥5.5°C)**, while **2.42 % (95 % eCI 0.05 % to 4.42 %) in winter were linked to DTR (≥8°C)** [39]. Lichtman et al. (2016) found that increased diurnal temperature variation (DTR) was associated with higher odds of stroke hospitalization in the **United States**, especially in the Northeast during spring to fall. Each **5°F increase in DTR correlated with a 5 %-37 % increase in hospitalization odds for those aged 18**–**64** and a **9 %-20 % increase for individuals aged 65 and older** [48]. Finally, He et al. (2021) reported a significant association between high DTR and ischemic stroke hospitalizations in Hefei, China [40], while Lee et al. (2010) reported a 6.8 % increase in acute myocardial infarction admissions linked to a 5 °C increase in DTR in Korea [41]. Lee et al. (2014) further identified seasonal sensitivity to DTR effects on MI visits in Korea, emphasizing the overall impact of temperature fluctuations on cardiovascular health [42].

### 4.1 Meta-analysis

Sixteen studies were eligible for the quantitative evaluation. Twelve sets of meta-analyses were performed to investigate the effects of diurnal temperature on hospital admission due to cardiovascular causes, including 1) sex, all age groups, and all cardiovascular causes (number of studies = 6); 2) sex and all age groups; heart failure (n = 3); 3) both sex and all age groups; stroke (n = 8); 4) both sex and all age groups; acute myocardial infarction (AMI) (n = 3); 5) both sex and elderly (≥65 years old); and all cardiovascular causes (n = 4); 6) both sexes and people aged <65 years old and all cardiovascular causes (n = 4); 7) females and all age groups and all cardiovascular causes (n = 4); 8) males and all age groups and all cardiovascular causes (n = 4); 9) both sex and elderly (≥65 years old); and 10) both sexes and people aged <65 years and stroke (n = 5), 11) females and all age groups, stroke (n = 5); and 12) males and all age groups, all age groups, and stroke (n = 5).

**Fig. 1:**
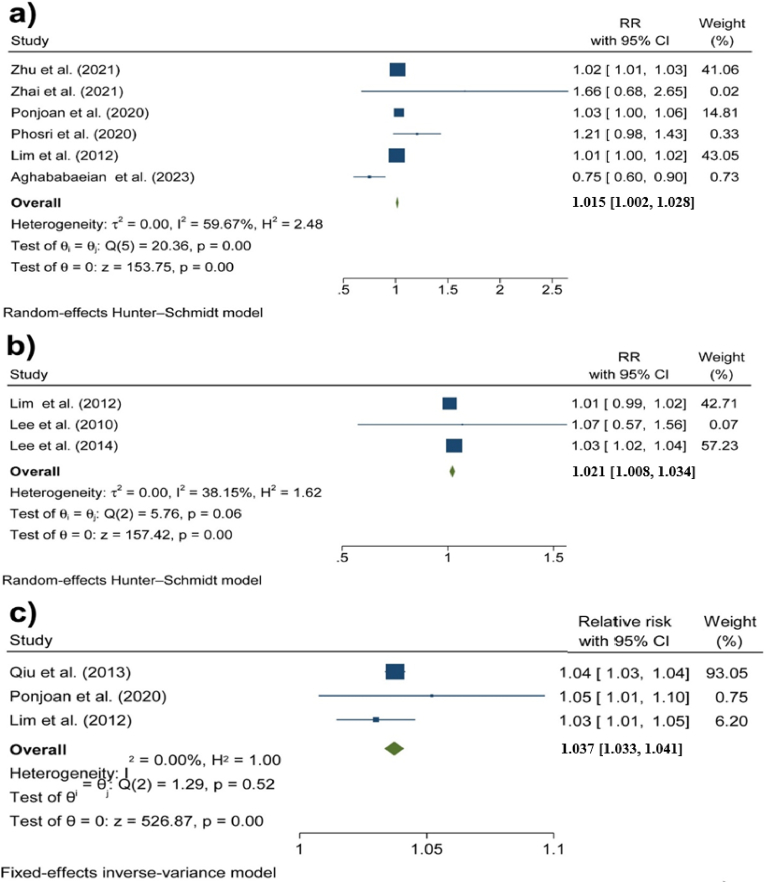
Associations between DTR and hospital admission due to adverse cardiovascular outcomes: a) all cardiovascular causes, b) AMI, and c) heart failure.

Fig. 1 illustrates the results of the meta-analyses conducted for the association between diurnal temperature range and hospital admission due to cardiovascular outcomes, including a) all cardiovascular causes, b) AMI, and c) heart failure. In the case of all cardiovascular causes, the heterogeneity between the studies was high enough to perform a random effects model (I^2^>50 %). In addition, funnel plots and Egger tests showed that there was no publication bias for any of the three sets of meta-analyses (P > 0.05) (see the Supplementary Materials).

The overall results of the meta-analysis indicated that a 1 °C increase in DTR was associated with a 1.5 % increase in all CVD hospital admissions (95 % CI: 0.2 %, 3 %). In addition, per 1 °C increase in DTR, hospital admission due to AMI and heart failure increased by RRs of 1.02 (95 % CI: 1.01, 1.03) and 1.04 (95 % CI: 1.03, 1.04), respectively (See Supplementary Materials 2).

**Fig. 2:**
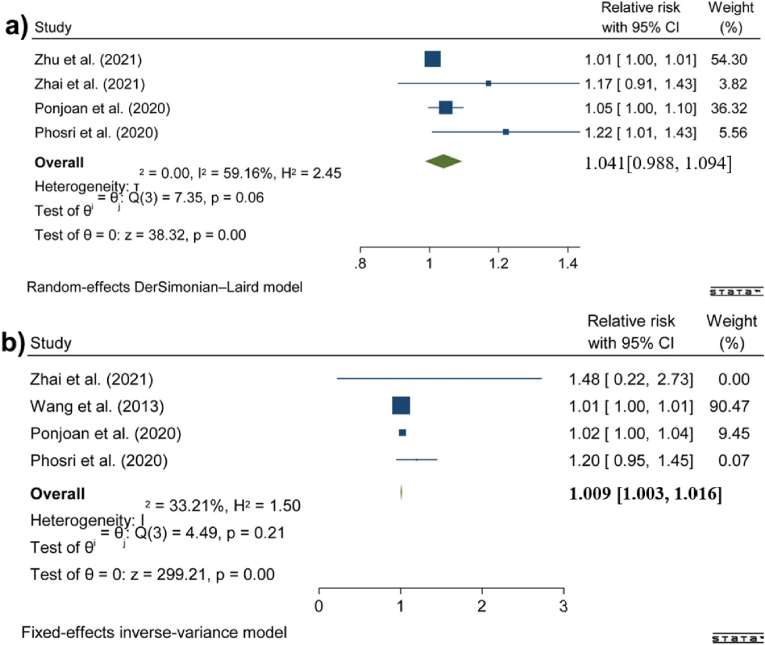
Associations between DTR and hospital admission due to all cardiovascular causes in a) people aged less than 65 years and b) people aged 65 years or older.

Fig. 2 shows the results of the meta-analyses implemented for the relationship between DTR and hospital admission due to all cardiovascular causes in people aged less than 65 years and people aged 65 years or older. High heterogeneity was found among the studies investigating people aged less than 65 years. Additionally, publication bias was found in the case of both sets of meta-analyses (see the Supplementary Materials). Our analysis showed that a 1 °C increase in DTR was associated with a 0.9 % increase in all CVD hospital admissions among those aged ≥65 years (95 % CI: 0.3 %, 1.6 %) (See Supplementary Materials 2).

**Fig. 3:**
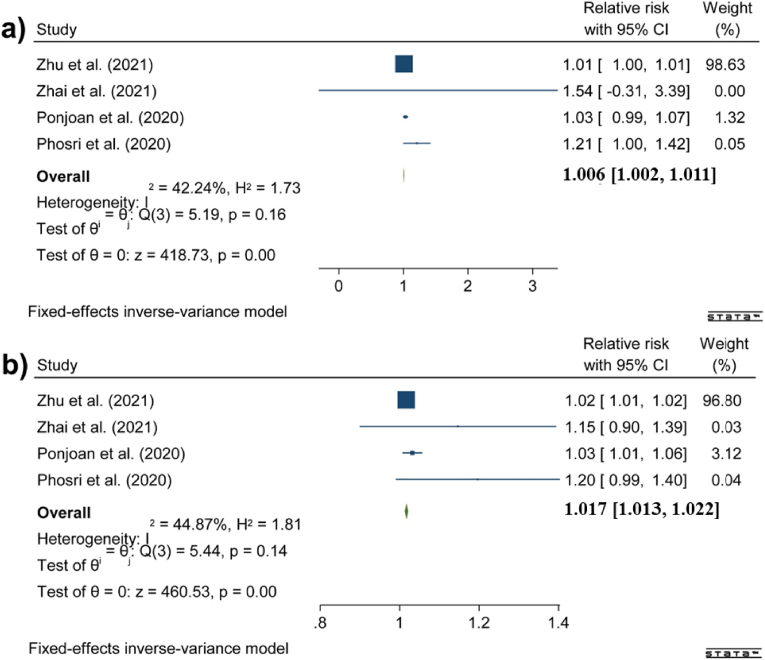
Associations between DTR and hospital admission due to all cardiovascular causes in a) females and b) males.

Fig. 3 presents the results of the meta-analyses for the association between DTR and hospital admission due to all cardiovascular causes in females and males. No heterogeneity was found among the studies. Additionally, publication bias was found in the case of both sets of meta-analyses (see the Supplementary Materials). The overall estimates indicated that DTR (per 1 °C increment) was associated with a 0.6 % (95 % CI: 0.2 %, 1.1 %) increase in females and a 1.7 % (95 % CI: 1.3 %, 2.2 %) increase in males, respectively (See Supplementary Materials 2).

**Fig. 4:**
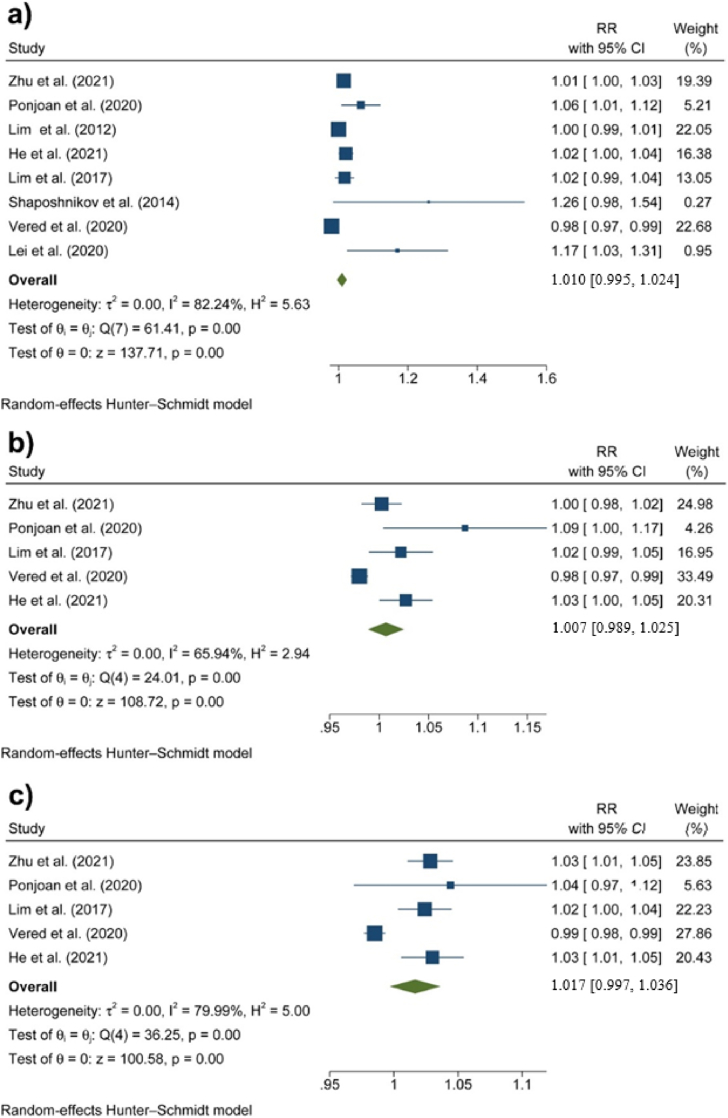
Associations between DTR and hospital admission due to stroke in a) the whole population, b) females, and c) males.

Fig. 4 displays the meta-analysis results concerning the impact of DTR on stroke incidence. The results of heterogeneity and publication bias tests are provided in the Supplementary Materials. An increase in DTR correlated with a general increase in hospital admissions due to stroke (RR: 1.010 [95 % CI: 0.995, 1.024]), but this association was not statistically significant. There was also no significant association between DTR and stroke in women (RR: 1.007 [95 % CI: 0.989, 1.025]), and similarly, there was no significant association in men (RR: 1.017 [95 % CI: 0.997, 1.036]), although the results were very close to significance. This finding suggests that fluctuations in DTR may affect hospital admissions for stroke, but the evidence is not statistically significant (See Supplementary Materials 2).

**Fig. 5:**
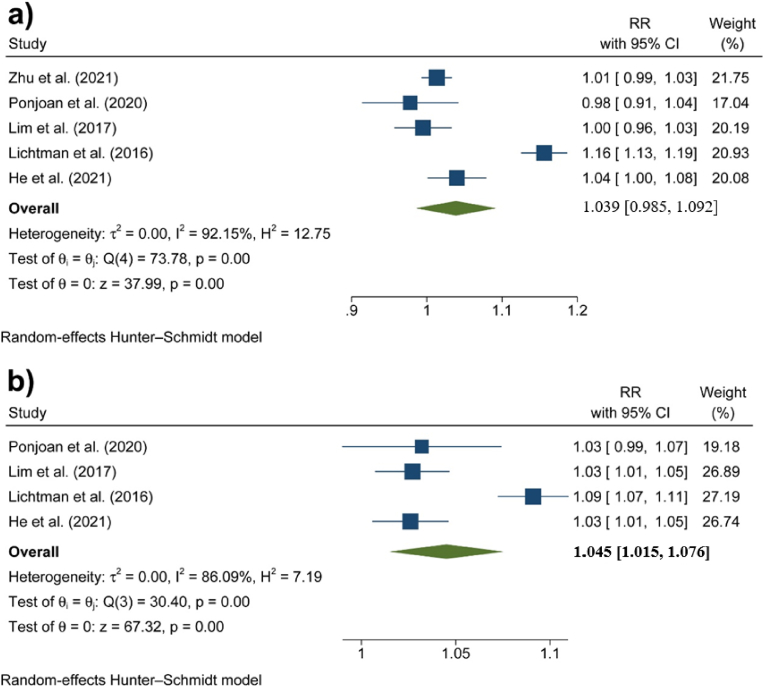
Associations between DTR and hospital admission due to stroke in a) people aged less than 65 years and b) people aged ≥65 years.

Fig. 5 presents forest plots illustrating the impact of DTR on hospital admissions for stroke in individuals aged less than 65 years and those aged more than 65 years. The results of heterogeneity and publication bias tests are provided in the Supplementary Materials. Although the association was not significant for the younger age group (RR: 1.039 [95 % CI: 0.985, 1.092]), it was statistically significant for elderly individuals, corresponding to a 4.5 % increase in stroke risk (RR: 1.045 [95 %: 1.015, 1.076]) (See Supplementary Materials 2).

**Flowchart 1:**
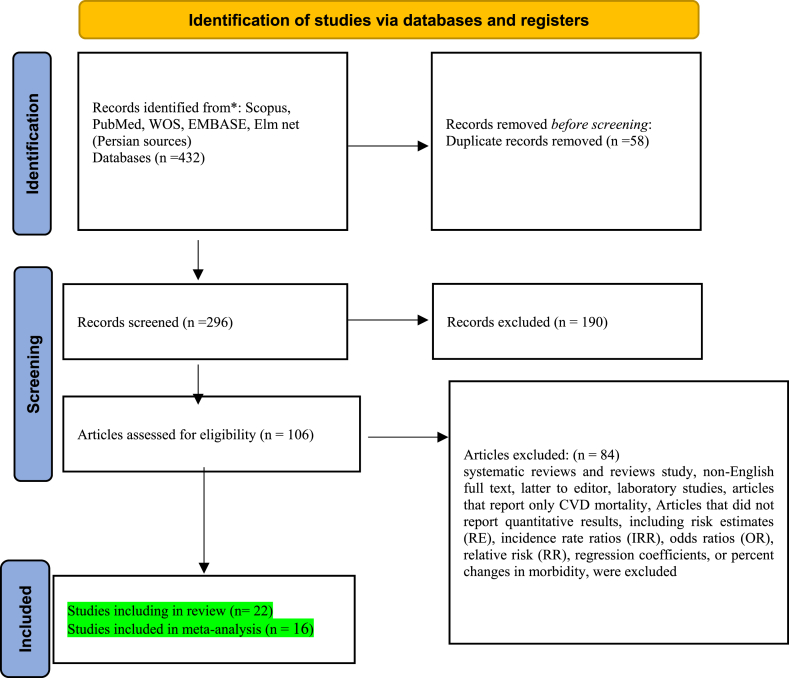
Identification of studies via databases and registers.

## 5 Discussion

Our meta-analysis revealed a significant association between the diurnal temperature range (DTR) and hospital admissions for CVD, with a 1 °C increase in DTR linked to a 1.5 % increase in overall CVD hospitalizations (RR: 1.015, 95 % CI: 1.002, 1.028). Additionally, we found that for acute myocardial infarction (AMI), a 1 °C increase in DTR is associated with a 2.1 % increase in hospital admissions (RR: 1.021, 95 % CI: [1.008, 1.034]), and for heart failure, there is a 3.7 % increase in admissions (RR: 1.037, 95 % CI: [1.033, 1.041]). These relationships underscore the potential role of environmental stressors, such as temperature variability, in exacerbating cardiovascular conditions. Previous research has highlighted how temperature fluctuations can significantly influence cardiovascular morbidity and mortality, emphasizing the need to consider these environmental factors in managing heart health. For instance, Lee et al. (2013) reported that both extreme temperatures and rapid temperature changes are associated with increased cardiovascular events [49]. The physiological mechanisms underlying these effects likely involve increased cardiac workload, destabilization of atherosclerotic plaques, and enhanced inflammatory responses [50,51]. Systemic stress induced by temperature variability may trigger adverse cardiovascular outcomes, especially in individuals with preexisting conditions. This finding is particularly pertinent in the context of global climate change, which is expected to increase the frequency and intensity of temperature fluctuations [52]. Therefore, public health strategies must consider the implications of DTR to mitigate the anticipated increase in cardiovascular events. Policies aimed at improving environmental resilience, such as urban planning to reduce heat islands and public education on coping mechanisms during extreme weather, could be crucial in managing the health impacts of DTR [53,54].

Our analysis revealed sex-specific differences in the association between DTR and hospital admissions for cardiovascular events. For instance, the risk of cardiovascular admission increased by 2 % in males (RR: 1.017, 95 % CI: 1.013, 1.022) compared to 1 % in females (RR: 1.006, 95 % CI: 1.002, 1.011). This disparity suggests that men might be more susceptible to the physiological impacts of temperature variability. Studies have shown that men generally have higher baseline risks for cardiovascular diseases due to factors such as a higher incidence of smoking and alcohol consumption, as well as differences in occupational exposures [55,56]. Additionally, men are more likely to engage in outdoor activities and strenuous physical work, which may increase their exposure to temperature extremes and fluctuations [57]. The physiological response to cold and heat stress also differs between sexes, with men exhibiting greater vasoconstriction and greater increases in blood pressure in response to cold exposure [58,59]. This could explain the greater sensitivity of men to DTR-related cardiovascular stress. Understanding these sex differences is crucial for developing targeted interventions. Health advisories and protective measures could be sex specific, with more emphasis on reducing exposure to temperature extremes for men, especially those with known cardiovascular risks. Additionally, workplace regulations could be adapted to minimize temperature-related risks in male-dominated occupations.

The impact of the DTR on hospital admissions for CVD also varies by age. Our findings revealed no significant increase in admissions for individuals under 65 years old (RR: 1.041, 95 % CI: 0.988, 1.094) compared to those aged 65 and older, who experienced a significant increase (RR: 1.009, 95 % CI: 1.003, 1.016). This differential effect might be attributed to the varying physiological responses to temperature changes across age groups. Younger individuals typically have more robust thermoregulatory mechanisms, but the stress induced by temperature variability can still exacerbate underlying cardiovascular conditions [60]. In contrast, older adults often have diminished physiological reserves and are more susceptible to environmental stressors, including temperature fluctuations. Aging is associated with reduced cardiac output, impaired vasodilation, and diminished capacity to sweat, all of which can impair the body's ability to cope with temperature changes [53,59]. Moreover, the prevalence of comorbidities such as hypertension, diabetes, and chronic obstructive pulmonary disease is greater in older populations, compounding their vulnerability [61]. Given these age-related differences, public health strategies must prioritize older adults in efforts to mitigate the health impacts of DTR. Interventions could include enhancing indoor climate control in residential settings for elderly individuals, increasing access to air conditioning, and providing community support services during periods of extreme weather. Additionally, healthcare providers should be aware of the heightened risks posed by temperature variability and incorporate this awareness into management plans for elderly patients with cardiovascular conditions.

Other findings from this study indicate that the diurnal temperature range (DTR) has a significant impact on the incidence of stroke-related hospital admissions. The results show that with an increase in DTR, the risk of hospitalization for elderly individuals with stroke significantly rises (RR: 1.045 [1.015, 1.076]), while the risk of total stroke hospitalization associated with increasing DTR, although increased, was not statistically significant (RR: 1.010 [0.995, 1.024]). In this regard, it seems that future studies could further clarify the role of DTR on total stroke admissions. Although considering age as a factor in the analysis of DTR impacts on stroke admissions adds another layer of depth to our understanding, it is essential to recognize how this factor interacts with the overall findings regarding DTR and stroke risk. The increased risk associated with older populations, as indicated by subgroup analyses, can be particularly concerning given the aging of the global demographic and the increased baseline vulnerability of these populations to cardiovascular diseases (CVDs). Elderly individuals often have compromised thermoregulatory mechanisms, which increase susceptibility to the adverse effects of temperature extremes [14,40]. These individuals are also more likely to have preexisting health conditions that could exacerbate their response to rapid temperature changes, thus increasing their risk of stroke during periods of high DTR [62]. Moreover, healthcare infrastructure needs to adapt to these findings by incorporating climate resilience into planning and response strategies, ensuring that elderly people have the necessary resources and support during adverse weather conditions [63,64].

On the other hand, in connection with the application of the GAM in most studies for data analysis, generalized additive models (GAMs) are recognized as powerful tools in ecological studies, particularly in public health research, allowing researchers to explore nonlinear and complex relationships between independent variables (such as temperature) and dependent variables (such as cardiovascular diseases). The flexibility of GAM enables the use of nonlinear functions, which is especially beneficial in ecological data where relationships may not be straightforward. Additionally, GAMs facilitate the analysis of temporal and spatial data, making them crucial for assessing environmental impacts on human health. By employing the GAM, researchers can effectively evaluate the risks associated with environmental changes and provide accurate predictions of future impacts [[65], [66], [67]].

## 6 Conclusion

The results of this meta-analysis revealed that changes in temperature, as measured by the daily temperature range (DTR), lead to an increase in hospitalizations for cardiovascular diseases. Cardiovascular diseases play a significant role in the health indicators and health economy of societies. Considering the importance of these diseases for productive and active groups in society, including adults, and the existing research on the effects of these diseases on vulnerable populations, such as elderly individuals and women, further investigation is necessary. Future studies should focus on exploring the causes behind the differences in study results, as well as developing intervention measures, planning and adaptation solutions, preventive actions, and strategies to reduce vulnerability to climate-related risks within the healthcare system. Given the substantial impact of DTR on the risk of cardiovascular disease hospitalizations, these findings underscore the need for policymakers and public health authorities to implement targeted interventions and adaptation measures to mitigate the adverse health effects associated with daily temperature fluctuations. Strengthening the resilience of healthcare systems and vulnerable populations to climate-related health risks should be a priority for promoting overall community well-being.

## CRediT authorship contribution statement

**Hamidreza Aghababaeian:** Writing – review & editing, Writing – original draft, Visualization, Validation, Supervision, Software, Resources, Project administration, Methodology, Investigation, Funding acquisition, Formal analysis, Data curation, Conceptualization. **Mostafa Hadei:** Visualization, Validation, Supervision, Software, Methodology, Formal analysis. **Mahsa Sepasian:** Writing – review & editing, Writing – original draft, Investigation, Formal analysis, Data curation, Conceptualization. **Masoumeh Gharaee:** Writing – review & editing, Writing – original draft, Visualization, Validation. **Ladan Araghi Ahvazi:** Writing – review & editing, Writing – original draft, Visualization, Validation. **Rahim Sharafkhani:** Writing – original draft, Software, Resources. **Mohammad Zarei:** Writing – review & editing, Writing – original draft, Data curation.

## Ethical approval

Ethics Code: IR.DUMS.SPH.REC.1399.

## Declaration of competing interest

The authors declare that they have no conflicts of interest.
