# Supplementary material for: Diurnal temperature range and hospital admission due to cardiovascular diseases: A systematic review and meta-analysis study
Source: Int J Cardiol Cardiovasc Risk Prev. 2025 Aug 15;27:200487. doi: 10.1016/j.ijcrp.2025.200487 (PMC12744817; doi:10.1016/j.ijcrp.2025.200487)
Supplement: Multimedia component 1 [file mmc1.docx]

| No. | References | Author(s) And Year | Study Region | Gender, Age | Research Design and Statistical Analysis | DTR Amounts | Outcome(S) | Key Findings | Effects Estimates |
| --- | --- | --- | --- | --- | --- | --- | --- | --- | --- |
|  | (1) | (Zhu et al., 2021) | Jinchan, Gansu province, (China) | Gender:  Male and Female  Age:  < 65, 65–74, ≥ 75 | GAM | Min = 1.80°C  Mean= 13.39°C  Max= 25.30°C | O&ER^[[1]](#footnote-1)^ admissions for CVDs | - A positive correlation between DTR and O&ER admissions of all CVDs. - Increase of DTR showed an increasing trend of all case studied diseases. - The cumulative lag effect was more obvious than the single lag effects. - Estimates of DTR on O&ER admissions of CVDs was higher among males and the elderly. - DTR has a more significant impact on O&ER admissions of CVDs in non-heating season than heating season - Effects of DTR on O&ER admissions with all CVDs, HTN^[[2]](#footnote-2)^ and stroke in non-heating season were higher than that in heating season, especially in cumulative lag days. | Single lag effect: Per 1 °C DTR increase,  CVDs: Lag3 increase 0.41% (0.28–0.55%)  HTN: Lag1 0.58% (0.38–0.79%)  IHD^[[3]](#footnote-3)^: Lag3 0.71% (0.38–1.03%)  Stroke: Lag3 0.76% (0.20–1.33%)  Cumulative lag effect: Per 1 °C DTR increase  CVDs: Lag07 increase 1.30% (0.99–1.62%)  HTN: Lag07 increase 1.73% (1.30–2.15%)  IHD: Lag04 1.71% (1.05–2.38 %-)  Stroke: Lag 07 1.49% (0.14–2.86%)  Male: Per 1 °C DTR increase  CVDs: 1.78% (1.36–2.21%)  HTN: 2.11% (1.55–2.67%)  Old (65–75 and ≥ 75): Per 1 °C DTR increase  CVDs: 1.80% (1.17–2.43%)  HTN: 1.80% (1.03–2.58%)  Heating seasons (single day lag):  CVDs: Lag1 0.47 (0.21–0.73)  HTN: Lag1 0.82 (0.48–1.17)  IHD: Lag4 0.77 (0.23–1.31)  Stroke: Lag3 0.36 (- 0.59 to 1.33)  Non-heating seasons (single day lag):  CVDs: Lag3 0.82 (0.65–1.00)  HTN: Lag3 0.73 (0.50–0.97)  IHD: Lag3 0.97 (0.56–1.39)  Stroke: Lag3 1.08 (0.37–1.79)  Heating seasons (cumulative day lag):  CVDs: Lag01 0.31 (- 0.02 to 0.64)  HTN: Lag01 0.79 (0.36–1.23)  IHD: Lag07 1.60 (0.11–3.11)  Stroke: Lag07 - 1.85 (- 4.41 to 0.79)  Non-heating seasons (cumulative day lag):  CVDs: Lag07 1.87 (1.45–2.30)  HTN: Lag07 2.01 (1.44–2.58)  IHD: Lag04 1.49 (0.64–2.34)  Stroke: Lag04 2.29 (0.85–3.75) |
|  | (2) | (Zheng et al., 2016) | Haidian District of Beijing, (china) | Gender:  Male and Female  Age:  65 and older | GAM | Min = 1.10°C  Mean= 9.85°C  Max= 21.90°C  SD= 3.58 | Daily ER Admissions for Cause-Specific CVD (all CVDs, HTN, IHD, and CeVD^[[4]](#footnote-4)^) | - ER admissions for cause-specific CVDs among the elderly was associated with short-term increase in DTR. - Acute health effects of DTR May increase or decrease by gender, age and season - The effects of DTR in females were higher than in males - A significant association was found for all cardiovascular disease ER admissions with DTR at lag 03 and 04 day among males only. | Single lag effect: Per 1 °C DTR increase,  Male:  CVDs:  Lag0 0.36 (-0.23~0.95) Lag1 0.42 (-0.18~1.03)  Lag2 0.41 (-0.18~1.00) Lag3 0.39 (-0.18~0.96)  Lag4 0.26 (-0.30~0.82)  HTN:  Lag0 -0.46 (-1.61~0.70) Lag1 0.16 (-1.04~1.38)  Lag2 0.19 (-0.99~1.39) Lag3 0.40 (-0.75~1.57)  Lag4 0.63 (-0.50~1.78)  IHD:  Lag0 -0.14 (-1.23~0.96) Lag1 0.88 (-0.24~2.01)  Lag2 0.82 (-0.26~1.91) Lag3 0.78 (-0.26~1.84)  Lag4 0.00 (-1.02~1.03)  CeVD:  Lag0 0.81 (-0.08~1.70) Lag1 0.61 (-0.32~1.54)  Lag2 0.49 (-0.41~1.40) Lag3 0.87 (-0.01~1.76) Lag4 0.41 (-0.46~1.29)  Female:  CVDs:  Lag0 1.01 (0.43~1.59)^*^ Lag1 0.90 (0.30~1.50)^*^  Lag2 0.14 (-0.45~0.73) Lag3 -0.27 (-0.84~0.30)  Lag4 -0.07 (-0.63~0.49)  HTN:  Lag0 0.83 (-0.15~1.83) Lag1 0.23 (-0.85~1.33)  Lag2 0.08 (-0.97~1.13) Lag3 -0.33 (-1.34~0.69)  Lag4 -0.22 (-1.21~0.79)  IHD:  Lag0 1.26 (0.22~2.31) ^*^ Lag1 0.79 (-0.28~1.86)  Lag2 0.47 (-0.57~1.52) Lag3 -0.38 (-1.39~0.64)  Lag4 -0.24 (-1.24~0.77)  CeVD:  Lag0 1.01 (0.06~1.97)^*^  Lag1 1.07 (0.09~2.07)^*^  Lag2 0.24 (-0.73~1.22) Lag3 0.18 (-0.77~1.13) Lag4 -0.21 (-1.15~0.73)  cumulative day lag: Per 1 °C DTR increase,  Male:  CVDs:  Lag01 0.61 (-0.13~1.36) Lag02 0.86 (-0.01~1.73)  Lag03 1.04 (0.09~2.00)^*^ Lag04 1.13 (0.11~2.16)^*^  HTN:  Lag01 -0.25 (-1.68~1.21)  Lag02 -0.09 (-1.75~1.60)  Lag03 0.19 (-1.64~2.06)  Lag04 0.59 (-1.39~2.62)  IHD:  Lag01 0.57 (-0.81~1.96)  Lag02 1.12 (-0.49~2.75)  Lag03 1.56 (-0.20~3.35)  Lag04 1.43 (-0.45~3.34)  CeVD:  Lag01 1.08 (´0.02~2.19)  Lag02 1.33 (0.06~2.61) ^*^  Lag03 1.95 (0.44~3.48) ^*^  Lag04 2.13 (0.51~3.77) ^*^  Female:  CVDs:  Lag01 1.46 (0.74~2.19) ^*^ Lag02 1.46 (0.62~2.31) ^*^  Lag03 1.16 (0.22~2.10) ^*^ Lag04 0.99 (-0.03~2.02)  HTN:  Lag01 0.68 (-0.67~2.05)  Lag02 -0.09 (-1.75~1.60)  Lag03 0.35 (-1.37~2.10)  Lag04 0.16 (-1.69~2.04)  IHD:  Lag01 1.55 (0.26~2.86) _*_  Lag02 1.71 (0.21~3.23) ^*^  Lag03 1.30 (-0.34~2.97)  Lag04 1.06 (-0.70~2.85)  CeVD:  Lag01 1.54 (0.37~2.73) ^*^  Lag02 1.55 (0.19~2.93) ^*^  Lag03 1.53 (0.03~3.04) ^*^  Lag04 1.29 (-0.31~2.92)  Age:  Male  65–74  CVDs: 1.33 (-0.24~2.92)  HTN: 0.88 (-0.85~2.64)  IHD: 1.08 (-1.47~3.70)  CeVD: 1.10 (-1.28~3.54)  ≥75  CVDs: 1.31 (0.11~2.52) ^*^  HTN: 0.65 (-0.85~2.17)  IHD: 2.17 (-0.04~4.44)  CeVD: 2.94 (0.90~5.01) ^*^  Female:  65–74:  CVDs: 0.74 (-0.29~1.78)  HTN: 0.22 (-1.15~1.61)  IHD: 1.84 (-0.22~3.94)  CeVD: 0.22 (-1.42~1.90)  ≥75:  CVDs: 2.23 (1.24~3.22) ^*^  HTN: 1.53 (0.11~2.98) ^*^  IHD: 1.85 (0.15~3.57) ^*^  CeVD: 3.22 (1.30~5.18) ^*^ |
|  | (3) | (Zhai, Zhang, et al., 2021) | Jiuquan, (China) | Gender:  Male and Female  Age: all | DLNM | Min =  3.50 *◦*C (DTR with minimum hospital admission risk )  Mean= 14.00 *◦*C  Max=  23.60 *◦*C | CVDs hospital admissions | - The impact of low DTR was significantly more harmful than high DTR. - The RR^[[5]](#footnote-5)^ values increased with the increase of DTRs and reached a peak about 11◦C. - Low DTR: was harmful for men and the elderly people (≥ 65 years old than women and adult (*<*65 years old) - High DTR: No significant differences in both gender and age subgroups | CVDs  hospital admissions  Highest cumulative risk:  Lag 0-14  RR: 2.190 (1.404, 3.416).  Men:  cumulative risk:  occurred at lag 0–3, increased and peaked at lag 0–14  RR: 2.299 (1.238, 4.273).  Adult (*<*65 years old): occurred at lag 0–3, increased and peaked at lag 0–14  RR: 1.794 (1.129, 2.851)  Women: no significant elevated cumulative risk throughout the whole lag period. |
|  | (5) | (Zhai, Qi, et al., 2021) | Dingxi, (China) | Gender: Male and Female  Age:  (< 65- ≥65) | DLNM | Min =  1.6 *◦*C  Mean= 11.76 *◦*C  Max=  26.2 *◦*C  SD= 4.74 | CVD hospital admissions | - At the relatively low DTR (6°C), the effect of DTR on CVD in men and younger patients is weaker than in females and older patients, - At extremely high DTR (19°C) it is stronger in men and younger peoples. | Extremely low DTR (4 °C)  Total  Lag 0 1.042 (1.006,1.08), Lag1 1.082 (1.010,1.160)  Lag 3 1.157 (1.017,1.315), Lag7 1.27 (1.017,1.585)  Lag 14 1.384 (0.953,1.828), Lag 21 1.17 (0.764,1.794)  Male  Lag 0 1.020 (0.980,1.061), Lag1 1.039 (0.962,1.121)  Lag 3 1.072 (0.93,1.236), Lag7 1.122 (0.877,1.435)  Lag 14 1.141(0.798,1.643), Lag 21 1.084 (0.675,1.741)  Female  Lag 0 1.051 (1.011,1.091), Lag1 1.1 (1.022,1.184)  Lag 3 1.192 (1.04,1.368), Lag7 1.342 (1.059,1.701)  Lag 14 1.438 (1.012,2.031), Lag 21 1.283 (0.811,2.028)  Adult  Lag 0 1.083 (1.03,1.139), Lag1 1.166 (1.058,1.286)  Lag 3 1.331 (1.109,1.597), Lag7 1.622 (1.182,2.226)  Lag 14 1.859 (1.161,2.975), Lag 21 1.629 (0.872,3.045)  Old  Lag 0 1.081 (1.024,1.14), Lag1 1.162 (1.048,1.289)  Lag 3 1.327 (1.094,1.61) , Lag7 1.64 (1.174,2.293)  Lag 14 2.034 (1.217,3.292), Lag 21 1.964 (1.014,3.801)  Extremely high DTR (19 °C)  Total  Lag 0 0.982 (0.946,1.019), Lag1 0.966 (0.899,1.038)  Lag 3 0.941 (0.823,1.076), Lag7 0.915 (0.727,1.152)  Lag 14 0.941 (0.675,1.312), Lag 21 1.060 (0.7,1.629)  Male  Lag 0 0.987 (0.948,1.029), Lag1 0.977 (0.903,1.057)  Lag 3 0.963 (0.831,1.116), Lag7 0.961 (0.746,1.238)  Lag 14 1.044 (0.725,1.503), Lag 21 1.267(0.797,2.012)  Female  Lag 0 0.981 (0.942,1.021), Lag1 0.964 (0.892,1.041)  Lag 3 0.936 (0.81,1.081), Lag7 0.901 (0.703,1.156)  Lag 14 0.906 (0.633,1.296), Lag 21 0.996 (0.632,1.569)  Adult  Lag 0 1.087 (0.985,1.203), Lag1 1.126 (1.000,1.334)  Lag 3 1.257 (1.002,1.624), Lag7 1.395 (1.010,1.986)  Lag 14 1.445 (0.916,2.463), Lag 21 1.586 (0.931,3.025)  Old  Lag 0 0.999 (0.949,1.052), Lag1 1.000 (0.904,1.105)  Lag 31.003 (0.832,1.209), Lag7 1.020 (0.737,1.412)  Lag 14 1.088 (0.672,1.762), Lag 21 1.212 (0.644,2.285) |
|  | (7) | (Wang et al., 2013) | Haidian district of Beijing, china | Gender:  Male and female  Age:  65 years and older | GAM | Min = 1.10*◦*C  Mean= 9.85*◦*C  Max=  21.90 *◦*C | CVD | - The study provides additional evidence DTR is an independent risk factor for ER admissions among elderly. - There is no significant associations between CVD admissions and DTR were found in single-day lag structures. - A 1 °C increase in the 3-day and 6-day moving average of DTR (lag 02 and lag 05) corresponded to a 0.76% (0.07%–1.46%) increase in cardiovascular ER admissions. | Per 1 °C DTR increase: Single-day lag 0 0.35 (-0.12 to 0.82)  1 0.36 (-0.09 to 0.82)  2 0.25 (-0.18 to 0.68)  3 0.07 (-0.34 to 0.48)  4 0.01 (-0.39 to 0.42)  5 -0.27 (-0.66 to 0.13)  6 -0.34 (-0.74 to 0.05)  7 0.11 (-0.28 to 0.51)  Multi-day lag 01 0.62 (0.01–1.23)  02 0.76 (0.07–1.46)  03 0.73 (-0.02 to 1.49)  04 0.67 (-0.12 to 1.47)  05 0.41 (-0.42 to 1.25)  06 0.14 (-0.72 to 1.02)  07 0.22 (-0.69 to 1.14) |
|  | 10  (8) | (Rowland et al., 2021) | New York State, USA | Gender: mostly male and female,  Adult (≥18 years) | Spearman correlations, time-stratified case-crossover design |  | Myocardial infarction (MI) | - Percentage change in MI rate for a change in DTR | Mean to 10th Percentile -1.31 (-2.03, -0.60)  Mean to 90th Percentile 1.22 (0.59, 1.85) |
|  | (9) | (Qiu et al., 2013) | Hong Kong, (China) | Gender:  Male and Female  Age: <65, 65–74, and ≥75 | GAM | Min =  0.7*◦*C  Mean=  4.00 *◦*C  Max=  12. 2 *◦*C | Emergency HF^[[6]](#footnote-6)^ hospital admissions | - Greater temperature change within a day was associated with increased HF emergency hospital admissions. - DTR showed a significant effect in the cold season and on female and elderly patients. - The effects of DTR were significantly greater in the cool season. | Per 1°C increase in DTR:  Lag0 0.87 (0.31 to 1.43)  Lag1 0.89 (0.34 to 1.43)  Lag2 0.81 (0.28 to 1.34) Lag3 0.86 (0.35 to 1.38)  Lag4 0.63 (0.12 to 1.14)  Lag5 0.63 (0.12 to 1.14)  Overall cumulative_:_ 3.76 (3.36 to 4.16  Men  Lag0 1.31 (0.60 to 2.02)^*^  Lag1 0.68 (-0.01 to 1.38)  Lag2 0.72 (0.05 to 1.40)  Lag3 1.15 (0.50 to 1.81)  Lag4 0.88 (0.23 to 1.54)  Lag5 0.75 (0.10 to 1.41)  Overall cumulative: 4.41 (3.89 to 4.92) ^*^  Women  Lag0 0.29 (-0.49 to 1.09)  Lag1 1.15 (0.38 to 1.93)  Lag2 0.93 (0.19 to 1.69)  Lag3 0.49 (-0.24 to 1.22)  Lag4 0.30 (-0.42 to 1.03)  Lag5 0.47 (-0.26 to 1.20)  Overall cumulative: 2.93 (2.37 to 3.50)  <65  Lag0 0.96 (-0.59 to 2.52)  Lag1 0.54 (-0.96 to 2.07)  Lag2 0.99 (-0.48 to 2.48)  Lag3 0.61 (-0.82 to 2.06)  Lag4 -0.09 (-1.50 to 1.34)  Lag5 -0.23 (-1.64 to 1.21)  Overall cumulative 2.18 (1.07 to 3.30)  65-74  Lag0 0.93 (-0.14 to 2.01)  Lag1 0.08 (-0.96 to 1.13)  Lag2 0.31 (-0.70 to 1.33)  Lag3 0.59 (-0.39 to 1.59)  Lag4 1.21 (0.23 to 2.20)  Lag5 0.97 (-0.01 to 1.97)  Overall cumulative: 3.29 (2.51 to 4.06)  ≥75  Lag0 0.83 (0.18 to 1.48)  Lag1 1.20 (0.56 to 1.84)  Lag2 0.94 (0.32 to 1.56)  Lag3 0.98 (0.38 to 1.58)  Lag4 0.54 (-0.06 to 1.13) Lag5 0.64 (0.04 to 1.24)  Overall cumulative: 4.13 (3.66 to 4.60) |
|  | (10) | (Ponjoan et al., 2021) | Mediterranean region | Gender:  Male and Female.  Age:  18 years and older | SCCS^[[7]](#footnote-7)^ | Mean=  14.1°C  SD=  7.8°C | CVD hospitalization | - Extreme high, but not extreme low DTR increased CVD hospitalizations when it has an increasing trend. - This effect was more severe on the day of sudden temperature change and hospitalization due to stroke and heart failure. | Extreme low DTR  Stroke  Same-day effect  IRR (95% CI)  All population  0.988 (0.936 to 1.043)  Men  0.994 (0.924 to 1.071)  Women  0.981 (0.907 to 1.062)  <65 years  0.994 (0.899 to 1.098)  ≥65 years  0.988 (0.926 to 1.053)  Cumulative effect  IRR (95% CI)  All population  0.988 (0.954 to 1.023)  Men 0.968 (0.922 to 1.016)  Women  1.011 (0.961 to 1.064)  <65 years  0.986 (0.924 to 1.052)  ≥65 years  0.989 (0.949 to 1.031)  HF  Same-day effect  All population  0.984 (0.942 to 1.028)  Men  1.027 (0.962 to 1.096)  Women  0.952 (0.898 to 1.009)  <65 years  0.998 (0.898 to 1.108)  ≥65 years  0.984 (0.938 to 1.032)  Cumulative effect  All population  0.998 (0.971 to 1.026)  Men  1.025 (0.982 to 1.069)  Women  0.978 (0.942 to 1.015)  <65 years  1.054 (0.986 to 1.127)  ≥65 years  1.054 (0.986 to 1.127)  CHD  Same-day effect  All population  1.001 (0.955 to 1.050)  Men  1.004 (0.948 to 1.064)  Women  0.995 (0.916 to 1.081)  <65 years  1.035 (0.966 to 1.109)  ≥65 years  0.973 (0.912 to 1.039)  Cumulative effect  All population  1.017 (0.986 to 1.048)  Men  1.006 (0.969 to 1.044)  Women  1.040 (0.986 to 1.095)  <65 years  1.022 (0.978 to 1.069)  ≥65 years  1.012 (0.971 to 1.055)  All CVD  Same-day effect  All population  0.991 (0.964 to 1.019)  Men  1.009 (0.972 to 1.048)  Women  0.970 (0.931 to 1.010)  <65 years  1.016 (0.966 to 1.068) ≥65 years  0.982 (0.950 to 1.015)  Cumulative effect  All population  1.002 (0.984 to 1.020)  Men  1.002 (0.978 to 1.027)  Women  1.001 (0.975 to 1.027)  <65 years  1.021 (0.988 to 1.054)  ≥65 years  0.994 (0.974 to 1.016)  Extreme high DTR  Stroke  Same-day effect  IRR (95% CI)  All population  1.064 (1.010 to 1.121) ^*^  Men  1.044 (0.971 to 1.121) Women  1.087 (1.008 to 1.173) ^*^  <65 years  1.083 (0.985 to 1.192)  ≥65 years  1.051 (0.987 to 1.119) Cumulative effect  IRR (95% CI)  All population  1.020 (0.986 to 1.056) Men  1.016 (0.969 to 1.066) Women  1.026 (0.976 to 1.079) <65 years  0.978 (0916 to 1.043) ≥65 years  1.032 (0.991 to 1.075)  HF  Same-day effect  All population  1.052 (1.009 to 1.098)^*^ Men  1.074 (1.008 to 1.146)^*^ Women  1.036 (0.979 to 1.096) <65 years  1.123 (1.017 to 1.242)^*^ ≥65 years  1.032 (0.984 to 1.081) Cumulative effect  All population  1.034 (1.006 to 1.063)^*^ Men  1.069 (1.025 to 1.115)^*^ Women  1.009 (0.972 to 1.046) <65 years  1.058 (0.990 to 1.131) ≥65 years  1.024 (0.993 to 1.055)  CHD  Same-day effect  All population  0.984 (0.939 to 1.033)  Men  0.996 (0.940 to 1.055)  Women  0.962 (0.885 to 1.046)  <65 years  0.995 (0.928 to 1.067)  ≥65 years  0.973 (0.911 to 1.039) Cumulative effect  All population  1.013 (0.983 to 1.044)  Men  1.012 (0.975 to 1.050)  Women  1.016 (0.963 to 1.071)  <65 years  1.006 (0.961 to 1.052)  ≥65 years  1.015 (0.974 to 1.058)  All CVD  Same-day effect  All population  1.032 (1.005 to 1.061) ^*^  Men  1.033 (0.996 to 1.072)  Women  1.031 (0.991 to 1.073)  <65 years  1.047 (0.997 to 1.099) ≥65 years  1.022 (0.989 to 1.055)  Cumulative effect  All population  1.023 (1.006 to 1.042) ^*^  Men  1.032 (1.007 to 1.056) ^*^  Women  1.015 (0.989 to 1.041)  <65 years  1.010 (0.978 to 1.044)  ≥65 years  1.023 (1.002 to 1.045)^*^ |
|  | (11) | (Phosri et al., 2020) | Bangkok, (Thailand) | Gender:  male and female  Age: all, <65 years, and ≥65 years | Quasi-Poisson generalized linear regression model combined with  DLNM | Min =  1.9*◦*C  Mean=  7.8 *◦*C  Max=  14. 1 *◦*C | CVDs hospital admissions | - Short-term exposure to extreme DTR was significantly associated with an increased risk of hospitalization for CVDs, especially during winter. - With 7.8 °C DTR as threshold value, the relative risk for CVDs hospital admission steadily increased above threshold. - the association between extremely high DTR on CVDs hospital admission was greater during winter compared to summer and rainy season. - the effect estimate for CVDs hospital admission did not significantly differ between males and females, as well as between young people and the elderly. | Cardiovascular All  Lag 0 1.051 (1.003, 1.101)  Lag 0–7 1.134 (1.022, 1.259)  Lag 0–14 1.202 (1.034, 1.398)  Lag 0–21 1.206 (1.002, 1.452) Male  Lag 0 1.044 (0.991, 1.101)  Lag 0–7 1.151 (1.022, 1.296)  Lag 0–14 1.197 (1.008, 1.421)  Lag 0–21 1.176 (0.953, 1.452)  Female  Lag 0 1.058 (1.004, 1.116)  Lag 0–7 1.115 (0.988, 1.257)  Lag 0–14 1.208 (1.014, 1.438)  Lag 0–21 1.239 (1.001, 1.535) < 65 years  Lag 0 1.041 (0.986, 1.098)  Lag 0–7 1.136 (1.007, 1.282)  Lag 0–14 1.221 (1.026, 1.454)  Lag 0–21 1.209 (0.976, 1.498)  ≥65 years  Lag 0 1.063 (1.009, 1.119)  Lag 0–7 1.129 (1.004, 1.269)  Lag 0–14 1.176 (0.992, 1.395)  Lag 0–21 1.198 (0.973, 1.475)  Whole year  Lag 0 1.051 (1.003, 1.101)  Lag 0–71.134 (1.022, 1.259)  Lag 0–14 1.202 (1.034, 1.398)  Lag 0–21 1.206 (1.002, 1.452) Winter  Lag 0 1.098 (0.974, 1.238)  Lag 0–7 1.336 (0.997, 1.791)  Lag 0–141.738 (1.068, 2.828)  Lag 0–21 1.937 (1.039, 3.611) Summer  Lag 0 0.980 (0.902, 1.065)  Lag 0–7 0.886 (0.679, 1.158)  Lag 0–14 0.784 (0.509, 1.209)  Lag 0–21 0.680 (0.387, 1.197) Rainy season  Lag 0 0.980 (0.925, 1.039)  Lag 0–7 0.971 (0.790, 1.193)  Lag 0–14 0.920 (0.673, 1.256)  Lag 0–21 0.896 (0.589, 1.364) |
|  | (12) | (Lim et al., 2012) | Seoul  Incheon Daegu  Busan  (South Korea) | Gender: Male and Female  Age:  all | Poisson GLM^[[8]](#footnote-8)^ and temperature-matched CC design | Seoul  Mean: 8  SD: 3  Incheon:  Mean:8  SD: 3  Daegu:  Mean:9  SD:3  Busan:  Mean: 7  SD: 2 | Hospital admissions of CVD (stroke, MI^[[9]](#footnote-9)^, IHD, CF^[[10]](#footnote-10)^, CD^[[11]](#footnote-11)^, ,arrhythmia | - A positive association between DTR and CVD hospital admissions. - The area-combined effects of DTR on some of the examined CVDs were statistically significant. - Among the diseases investigated in this study, the risk of cardiac failure was greatest in GLM and CC analyses, | Total cardiovascular  Seoul:  GLM 0.8 (−0.2,1.9)  CC −0.2 (−0.6,0.2)  Incheon:  GLM 1.0 (−0.5,2.5)  CC 0.3 (−0.6,1.1)  Daegu:  GLM 0.9 (−0.1,2.0)  CC 0.2 (−0.4,0.9)  Busan:  GLM 1.3 (−0.2,2.7)  CC −0.3 (−1,0.4)  Stroke  Seoul:  GLM 0.2 (−0.9,1.4)  CC 0.7 (−0.3,1.6)  Incheon:  GLM 0.2 (−1.8,2.2)  CC −0.7 (−2.6,1.2)  Daegu:  GLM −0.5 (−1.9,0.9)  CC 0.7 (−0.6,2.0)  Busan:  GLM 0.4 (−1.4,2.2)  CC 0.4 (−1.2,2.0)  MI  Seoul:  GLM 1.0 (−0.9,3.0)  CC 1.6 (−0.5,3.7)  Incheon:  GLM −1.6 (−5.8,2.7)  CC −2.9 (−9.1,3.8)  Daegu:  GLM 0.9 (−2.0,3.9)  CC −1.6 (−4.9,1.8)  Busan:  GLM 2 (−1.4,5.4)  CC −3.4 (−6.9,0.2)  IHD  Seoul:  GLM 0.4 (−1.1,1.8)  CC −1.5 (−2.5,−0.5)^⁎^  Incheon:  GLM 1.6 (−0.9,4.2)  CC 0.6 (−1.7,2.9)  Daegu:  GLM −1.1 (−2.9,0.8)  CC −1.3 (−3.1,0.5)  Busan:  GLM 1.0 (−1.1,3.2)  CC −0.5 (−2.2,1.2)  CF  Seoul:  GLM 2.4 (0.2,4.7) ^⁎^  CC 2.0 (−0.2,4.3)  Incheon:  1.5 (−2.9,6.1)  5.0 (−1.7,12)  Daegu:  5.0 (1.5,8.5) ^⁎^  13 (6.4,19.9)^⁎^  Busan:  3.2 (−0.3,6.8)  3.4 (−0.4,7.4)  CD  Seoul:  GLM 0.6 (−0.6,1.8)  CC −0.6 (−1.1,0.0) ^⁎^  Incheon:  GLM 0.8 (−0.9,2.4)  CC 1.4 (0.2,2.7) ^⁎^  Daegu:  GLM 0.2 (−1.1,1.4)  CC −0.3 (−1.2,0.7)  Busan:  GLM 0.8 (−0.8,2.4)  CC −0.9 (−1.8,0.1)  Arrhythmia  Seoul :  GLM 0 (−1.9,1.9)  CC −1.3 (−3.1,0.5)  Incheon:  GLM 2.4 (−1.7,6.6)  CC 19.9 (11.1,29.4) ^⁎^  Daegu:  GLM 3.9 (0.9,6.9) ^⁎^  CC 0.7 (−4.0,5.6)  Busan:  GLM 0.2 (−3.2,3.8)  CC −1.2 (−4.8,2.5) |
|  | (13) | Aghababaeian, H., et al. (2023). | Dezful, Iran | Gender: Male and Female  Age:  all | Distributed lag nonlinear model combined with a quasi-Poisson regression | Mean= 15.95  Min=1.1  Max=27.6  Full year  99th (24.6)  90th (21.4)  10th (9.3)  1 h (3.6)  Warm season  99th (25.3)  90th (22.4)  10th (14)  1 h (6.8)  cold seasons  99th (21.2)  90th (18.6)  10th (7.3)  1 h (3.2) | Cardiovascular admission | - In extreme low DTRs, the cumulative effects of cardiovascular admissions significantly increased in total, and in warm and cold seasons - In extreme high DTRs, the cumulative effects of cardiovascular significantly decreased | In extreme low DTRs, CER  Total (1th, 10th)  1th /Lag0–21, 1.69 (1.06–2.70) , P ≤ 0.05))  Warm season  1th / (Lag0–21, 1.35 (1.07–1.71) P ≤ 0.05).  cold seasons  1th /(Lag0-21, 1.74 (1.03–2.93), P ≤ 0.05))  10th /(Lag0-21, 1.31 (1.04–1.64), P ≤ 0.05))  In extreme high DTRs, CER  Total (99^th^. 90th)  99^th/^ (Lag0–13 0.75 (0.62–0.92)  99^th^ /Lag0–21, 0.63 (0.50–0.79) P ≤ 0.05),  In warm seasons  99^th^ /(Lag0–13, 0.70(0.55–0.89) P ≤ 0.05)  99th /(Lag0–21, 0.55 (0.41–0.74) P ≤ 0.05)  In cold seasons  99^th^ / (Lag0 0.98(0.96–0.99, P ≤ 0.05).  90^th^ / (Lag0 0.98 (0.97–0.99), P ≤ 0.05). |
|  | (14) | He, Y., et al. (2021) | Hefei, China | Gender: Male and Female  Age:  all | Generalized additive model combined  with distributed lag non-linear model | Daily mean ± SD  8.5 ± 3.8  Min= 1  Medium=8.3  MAX=21.4 | Hospitalizations for ischemic stroke | - High DTR was associated with hospitalizations for ischemic stroke. - There was a significant interactive effect of high DTR on ischemic stroke. | DTR (99th percentile [17.1 C])  Lag 8 RR 1.021, (1.002, 1.041)  Lag 12 RR 1.029, (1.011, 1.046) |
|  | (15) | Lee, J. H., et al. (2010) | Korea | Gender: Male and Female  Age:  all | Generalized additive Poisson models | - | hospital admissions for acute myocardial infarction | - There was a negative correlation between daily Acute Myocardial Infarction hospital admissions and diurnal temperature range. - A 5 °C increase in diurnal temperature range was associated with a 6.8% increase in the number of hospital admissions (β=0.066, risk ratio=1.068, - p=0.04). | Diurnal temperature range (°C) (increased by 5 °C) 1.068 (0.066±0.032) P=0.04 |
|  | (16) | Lee, S., et al. (2014) | Korea | Gender: Male and Female  Age:  all | Poisson generalized additive model | Mean (SD)  8.77 (3.35) | Acute Myocardial Infarction | - The DTR above 7.5 or 8.5uC in the spring and autumn showed threshold effects for increased MI visits. Non-STEMI patients in the spring showed an - Increased risk for MI above 6.5uC of the DTR. The threshold temperature of DTR in the winter was 4.5 to 6.5uC, which was lower than in the spring or autumn. - Lag-day effects for DTR were 1 or 2 days; however, delayed lag effects were evident in males at 7 days and in the old age group at 4 days in winter. | Spring  Threshold(C(7.5  All Lag1 RR 1.03 (1.02–1.04)  Male Lag1 RR 1.03 (1.02–1.04)  Female Lag1 RR 1.04 (1.03–1.06)  Lower 75 years Lag1 RR 1.03 (1.02–1.05  Bigger 75 years Lag1 RR 1.03 (1.01–1.05)  Autumn  Threshold(C(8.5  Female Lag2 RR 1.04 (1.01–1.06)  Threshold(C(7.5  Bigger 75 years Lag1 RR 1.03 (1.01–1.06)  Winter  Threshold(C(6.5  All Lag1 RR1.02 (1.01–1.03)  Male Lag1 RR 1.02 (1.01–1.03)  Female Lag4 RR 1.03 (1.01–1.05)  Threshold(C(5.5)  Lower 75 years Lag1 RR 1.02 (1.01–1.04)  Threshold(C(6.5  Bigger 75 years Lag1 RR 1.02 (1.00–1.05) |
|  | (17) | Lei, L., et al. (2020) | Shenzhen, China | Gender: Male and Female  Age:  all | quasi-Poisson  regression  DLNM | Mean= 6.0  Min=1.2  Max= 14 | strokes | - High DTR had a significant association with first-ever strokes | High DTR (5.5°C and higher) in the summer  Total, 3.65% (1.81% to 5.53%)  High DTR (8°C and higher) in the winter.  2.42% (0.05% to 4.42%) |
|  | (18) | Lichtman, J. H., et al. (2016). | United States | Gender: Male and Female  Age: adults aged 18 years or older | logistic regression model | - | stroke | - Increased diurnal temperature variation was associated with increased odds of stroke hospitalization for nearly all regions in the spring to fall seasons | Each 5°F increase in DTR  Aged 18-64 years  In the winter  ORs 1.05,.99-1.12  In the spring  1.37, 1.29-1.46)    Aged 65 years or older  In the winter  ORs 1.09, 1.05-1.13  In the spring  1.20, 1.16-1.25). |
|  | (19) | Lim, J.-S., et al. (2017) | Korea | Gender: Male and Female  Age:  all | multiple Poisson regression | - | Stroke | - Diurnal temperature changes over the preceding 24 hours had significant associations with the incidences of ischemic stroke and any stroke. | DTR  Male  A 1°C change in temperature conferred a 2.4% higher risk for acute stroke. Male 1.024*  (1.004–1.045)  in older than 65 years of age, a 1°C change in temperature was associated with a 2.7% higher risk of acute ischemic stroke (RR 1.027, 1.008–1.047). |
|  | (20) | Mizuno, A., et al. (2015) | Tokyo/ Japan | Gender: Male and Female  Age:  all | Generalized additive Poisson regression | Diurnal temperature range Event days 6.3±2.2  Non Event days  6.1±2.2 | Pulmonary  Embolism | - Every 1C increase in the DTR at lag0 corresponded to an increased relative risk of hospital admission for PE | [OR] lag0 1.036, (1.003 to 1.070). |
|  | (21) | Shaposhnikov, D., et al. (2014). | Moscow, Russia | Gender: Male and Female  Age:  all | Poisson  generalized linear model | Diurnal temperature range (°C)  Model for MI  1 6 16  Model for BS (brain stroke)  1 7 16 | Acute myocardial infarction and brain stroke | - A statistically significant relationship with DTR was established only for BS, i.e., a J-shape with the minimum near DTR=4 °C | A 10 °C increase in DTR from 4 to 14 °C was associated with a 26 % (2 % to 57 %) increase in BS, with a peak lag of 5 days. |
|  | (22) | Yoneyama, K., et al. (2021) | Japan | Gender: Male and Female  aged ≤ 64 years | multilevel mixed-effect models | Diurnal weather temperature range, mean ± standard deviation℃ 7.7 ± 3.1 | cardiovascular diseases | - diurnal weather temperature range (DTR) was associated with a greater number of CVD hospitalizations | coefficient, 4.540 [4.310 to 4.765] per ℃, p < 0.001) |
|  | (23) | Vered, S., et al. (2020) | Israel | Gender: Male and Female  aged  50 and above, | conditional logistic regression analysis | DTR (°C)  Mean ± SD  9.9 ± 3.6  Median  9.7  Range 0.9–24.9 | stroke or transient ischemic attack: | - A decrease in stroke/TIA risk was detected with larger DTR | ICH Ischemic stroke/TIA  Age 50–74  0.94(0.88–1.01) 0.96(0.94–0.97)  >74  0.99(0.93–1.05) 0.97(0.95–0.98)  Men  0.96(0.90–1.02) 0.97(0.95–0.98)  Women  0.98(0.91–1.05) 0.95(0.94–0.97) |
|  | (24) | Zhai, G., et al. (2022) | Northwest China | Gender: Male and Female  Aged  Farmer  50 and above, | distributed lag non-linear model (DLNM) | DTR (°C)  Mean= 10.65  Min= 1  Max=24.3 | cerebrovascular disease | - A U-shape relationship was observed between DTR and hospital admissions for CVD. | When the lag period was 0–21 days, the impact of high  DTR (1.595 [95% CI 1.301–1.957]) was slightly more significant than that of a low DTR (1.579 [95% CI  − 1.202 to 2.075]).  Cumulative relative risks  Lag Low DTR High DTR  0 0.919 (0.864, 0.978) 0.920 (0.868, 0.974)  0–3 0.922 (0.829, 1.026) 0.865 (0.788, 0.949)  0–7 0.965 (0.832, 1.119) 0.946 (0.837, 1.070)  0–14 1.172 (0.952, 1.443) 1.028 (0.872, 1.213)  0–21 1.579 (1.202, 2.075) 1.595 (1.301, 1.957  Lag 0 / Lag 0–3 /Lag 0–7 / Lag 0–14/ Lag 0–21  Male  0.860 (0.790, 0.935)/ 0.903 (0.782, 1.043)/ 0.913 (0.748, 1.114)/ 1.120 (0.848, 1.480)/ 1.813 (1.259, 2.611)  Female  0.996 (0.908, 1.092)/ 0.942 (0.803, 1.105)/ 1.028 (0.823, 1.283)/ 1.231 (0.900, 1.684)/ 1.308 (0.867, 1.974) |

Table1 shows that all studies somehow indicated an increased risk of cardiovascular disease hospitalization following DTR changes. (Table 1).

1. Zhu W, Wei X, Zhang L, Shi Q, Shi G, Zhang X, et al. The effect and prediction of diurnal temperature range in high altitude area on outpatient and emergency room admissions for cardiovascular diseases. International archives of occupational and environmental health. 2021;94(8):1783-95.

2. Zheng S, Wang M, Li B, Wang S, He S, Yin L, et al. Gender, Age and Season as Modifiers of the Effects of Diurnal Temperature Range on Emergency Room Admissions for Cause-Specific Cardiovascular Disease among the Elderly in Beijing. International journal of environmental research and public health. 2016;13(5).

3. Zhai G, Zhang K, Zhou W, Wu T. Impact of diurnal temperature range on hospital admissions for cardiovascular disease in Jiuquan, China. Urban Climate. 2021;38.

4. Zhai G, Qi J, Zhang X, Zhou W, Wang J. A comparison of the effect of diurnal temperature range and apparent temperature on cardiovascular disease among farmers in Qingyang, Northwest China. Environmental science and pollution research international. 2022;29(19):28946-56.

5. Zhai G, Qi J, Chai G. Impact of diurnal temperature range on cardiovascular disease hospital admissions among Chinese farmers in Dingxi (the Northwest China). BMC cardiovascular disorders. 2021;21(1):252.

6. Zha Q, Chai G, Zhang ZG, Sha Y, Su Y. Effects of diurnal temperature range on cardiovascular disease hospital admissions in farmers in China's Western suburbs. Environmental science and pollution research international. 2021;28(45):64693-705.

7. Wang MZ, Zheng S, He SL, Li B, Teng HJ, Wang SG, et al. The association between diurnal temperature range and emergency room admissions for cardiovascular, respiratory, digestive and genitourinary disease among the elderly: a time series study. The Science of the total environment. 2013;456-457:370-5.

8. Rowland ST, Parks RM, Boehme AK, Goldsmith J, Rush J, Just AC, et al. The association between ambient temperature variability and myocardial infarction in a New York-State-based case-crossover study: An examination of different variability metrics. Environ Res. 2021;197:111207.

9. Qiu H, Yu IT, Tse LA, Tian L, Wang X, Wong TW. Is greater temperature change within a day associated with increased emergency hospital admissions for heart failure? Circulation Heart failure. 2013;6(5):930-5.

10. Ponjoan A, Blanch J, Alves-Cabratosa L, Martí Lluch R, Comas-Cufí M, Parramon D, et al. Extreme diurnal temperature range and cardiovascular emergency hospitalisations in a Mediterranean region. Occupational and environmental medicine. 2021;78(1):62-8.

11. Phosri A, Sihabut T, Jaikanlaya C. Short-term effects of diurnal temperature range on hospital admission in Bangkok, Thailand. The Science of the total environment. 2020;717:137202.

12. Lim YH, Hong YC, Kim H. Effects of diurnal temperature range on cardiovascular and respiratory hospital admissions in Korea. The Science of the total environment. 2012;417-418:55-60.

13. Aghababaeian H, Sharafkhani R, Kiarsi M, Mehranfar S, Moosavi A, Araghi Ahvazi L, et al. Diurnal temperature range and hospital admission due to cardiovascular and respiratory diseases in Dezful, a city with hot climate and high DTR fluctuation in Iran: an ecological time-series study. Environmental geochemistry and health. 2023:1-13.

14. He Y, Tang C, Liu X, Yu F, Wei Q, Pan R, et al. Effect modification of the association between diurnal temperature range and hospitalisations for ischaemic stroke by temperature in Hefei, China. Public Health. 2021;194:208-15.

15. Lee JH, Chae SC, Yang DH, Park HS, Cho Y, Jun J-E, et al. Influence of weather on daily hospital admissions for acute myocardial infarction (from the Korea Acute Myocardial Infarction Registry). International journal of cardiology. 2010;144(1):16-21.

16. Lee S, Lee E, Park MS, Kwon BY, Kim H, Jung DH, et al. Short-term effect of temperature on daily emergency visits for acute myocardial infarction with threshold temperatures. PLoS One. 2014;9(4):e94070.

17. Lei L, Bao J, Guo Y, Wang Q, Peng J, Huang C. Effects of diurnal temperature range on first-ever strokes in different seasons: a time-series study in Shenzhen, China. BMJ open. 2020;10(11):e033571.

18. Lichtman JH, Leifheit-Limson EC, Jones SB, Wang Y, Goldstein LB. Average temperature, diurnal temperature variation, and stroke hospitalizations. Journal of Stroke and Cerebrovascular Diseases. 2016;25(6):1489-94.

19. Lim J-S, Kwon H-M, Kim S-E, Lee J, Lee Y-S, Yoon B-W. Effects of temperature and pressure on acute stroke incidence assessed using a Korean nationwide insurance database. Journal of Stroke. 2017;19(3):295.

20. Mizuno A, Takeuchi A, Yamamoto T, Tanabe Y, Obayashi T, Takayama M, et al. Seasonal changes in hospital admissions for pulmonary embolism in metropolitan areas of Tokyo (from the Tokyo Cardiovascular Care Unit Network). The American journal of cardiology. 2015;116(12):1939-43.

21. Shaposhnikov D, Revich B, Gurfinkel Y, Naumova E. The influence of meteorological and geomagnetic factors on acute myocardial infarction and brain stroke in Moscow, Russia. International journal of biometeorology. 2014;58:799-808.

22. Yoneyama K, Nakai M, Higuma T, Teramoto K, Watanabe M, Kaihara T, et al. Weather temperature and the incidence of hospitalization for cardiovascular diseases in an aging society. Scientific reports. 2021;11(1):10863.

23. Vered S, Paz S, Negev M, Tanne D, Zucker I, Weinstein G. High ambient temperature in summer and risk of stroke or transient ischemic attack: a national study in Israel. Environmental Research. 2020;187:109678.

24. Zhai G, Zhang J, Zhang K, Chai G. Impact of diurnal temperature range on hospital admissions for cerebrovascular disease among farmers in Northwest China. Scientific Reports. 2022;12(1):15368.

1. Outpatient and Emergency Room [↑](#footnote-ref-1)
2. Hypertension [↑](#footnote-ref-2)
3. Ischemic Heart Disease [↑](#footnote-ref-3)
4. Cerebrovascular diseases [↑](#footnote-ref-4)
5. Relative Risk [↑](#footnote-ref-5)
6. Heart Failure [↑](#footnote-ref-6)
7. Self-Controlled Case Series [↑](#footnote-ref-7)
8. Generalized Linear Model [↑](#footnote-ref-8)
9. Myocardial Infarction [↑](#footnote-ref-9)
10. Cardiac Failure [↑](#footnote-ref-10)
11. Cardiac Disease [↑](#footnote-ref-11)
