# Supplementary material for: Diurnal temperature range and hospital admission due to cardiovascular diseases: A systematic review and meta-analysis study
Source: Int J Cardiol Cardiovasc Risk Prev. 2025 Aug 15;27:200487. doi: 10.1016/j.ijcrp.2025.200487 (PMC12744817; doi:10.1016/j.ijcrp.2025.200487)
Supplement: Multimedia component 2 [file mmc2.docx]

**Supplementary Materials**

**Results of meta-analysis**

**Both gender-All ages-CVD**

. meta set central lower upper, civartolerance(1) random(hschmidt) studylabel(authoryear) studysize(samplesize) eslabel(RR)

Meta-analysis setting information

Study information

No. of studies: 6

Study label: authoryear

Study size: samplesize

Effect size

Type: <generic>

Label: RR

Variable: central

Precision

Std. err.: _meta_se

CI: [_meta_cil, _meta_ciu]

CI level: 95%, controlled by level()

User CI: [lower, upper]

User CI level: 95%, controlled by civarlevel()

Model and method

Model: Random effects

Method: Hunter–Schmidt

. meta summarize

Effect-size label: RR

Effect size: central

Std. err.: _meta_se

Study label: authoryear

Meta-analysis summary Number of studies = 6

Random-effects model Heterogeneity:

Method: Hunter–Schmidt tau2 = 0.0001

I2 (%) = 59.67

H2 = 2.48

------------------------------------------------------------------------------

Study | RR [95% conf. interval] % weight

----------------------------+-------------------------------------------------

Zhu et al. (2021) | 1.018 1.010 1.026 41.06

Zhai et al. (2021) | 1.663 0.676 2.650 0.02

Ponjoan et al. (2020) | 1.032 1.004 1.060 14.81

Phosri et al. (2020) | 1.206 0.981 1.431 0.33

Lim et al. (2012) | 1.010 1.003 1.016 43.05

Aghababaeian et al. (2023) | 0.750 0.600 0.900 0.73

----------------------------+-------------------------------------------------

theta | 1.015 1.002 1.028

------------------------------------------------------------------------------

Test of theta = 0: z = 153.75 Prob > |z| = 0.0000

Test of homogeneity: Q = chi2(5) = 20.36 Prob > Q = 0.0011

**Heterogeneity**

. •meta galbraith

•meta is not a valid command name

r(199);

. meta galbraith

Effect-size label: RR

Effect size: central

Std. err.: _meta_se

Model: Common effect

Method: Inverse-variance

. meta bias, egger

Effect-size label: RR

Effect size: central

Std. err.: _meta_se

Regression-based Egger test for small-study effects

Random-effects model

Method: Hunter–Schmidt

H0: beta1 = 0; no small-study effects

beta1 = 0.08

SE of beta1 = 0.573

z = 0.14

Prob > |z| = 0.8860

.

**Both gender-All ages -Heart Failure**

.

. meta summarize, fixed(invvariance)

Effect-size label: Relative risk

Effect size: RR

Std. err.: _meta_se

Study label: Author

Meta-analysis summary Number of studies = 3

Fixed-effects model Heterogeneity:

Method: Inverse-variance I2 (%) = 0.00

H2 = 1.00

------------------------------------------------------------------------

Study | Relative risk [95% conf. interval] % weight

----------------------+-------------------------------------------------

Qiu et al. (2013) | 1.038 1.034 1.042 93.05

Ponjoan et al. (2020) | 1.052 1.007 1.097 0.75

Lim et al. (2012) | 1.030 1.015 1.045 6.20

----------------------+-------------------------------------------------

theta | 1.037 1.033 1.041

------------------------------------------------------------------------

Test of theta = 0: z = 526.87 Prob > |z| = 0.0000

Test of homogeneity: Q = chi2(2) = 1.29 Prob > Q = 0.5242

.

. meta bias, egger fixed

Effect-size label: Relative risk

Effect size: RR

Std. err.: _meta_se

Regression-based Egger test for small-study effects

Fixed-effects model

Method: Inverse-variance

H0: beta1 = 0; no small-study effects

beta1 = -0.07

SE of beta1 = 0.874

z = -0.08

Prob > |z| = 0.9392

**Both genders-All ages -Stroke**

. meta set central lower upper, civartolerance(1) random(hschmidt) studylabel(authoryear) studysize(samplesize) eslabel(RR)

Meta-analysis setting information

Study information

No. of studies: 8

Study label: authoryear

Study size: samplesize

Effect size

Type: <generic>

Label: RR

Variable: central

Precision

Std. err.: _meta_se

CI: [_meta_cil, _meta_ciu]

CI level: 95%, controlled by level()

User CI: [lower, upper]

User CI level: 95%, controlled by civarlevel()

Model and method

Model: Random effects

Method: Hunter–Schmidt

. meta summarize

Effect-size label: RR

Effect size: central

Std. err.: _meta_se

Study label: authoryear

Meta-analysis summary Number of studies = 8

Random-effects model Heterogeneity:

Method: Hunter–Schmidt tau2 = 0.0002

I2 (%) = 82.24

H2 = 5.63

-----------------------------------------------------------------------------

Study | RR [95% conf. interval] % weight

---------------------------+-------------------------------------------------

Zhu et al. (2021) | 1.015 1.001 1.028 19.39

Ponjoan et al. (2020) | 1.064 1.008 1.120 5.21

Lim et al. (2012) | 1.000 0.992 1.008 22.05

He et al. (2021) | 1.021 1.002 1.041 16.38

Lim et al. (2017) | 1.017 0.990 1.043 13.05

Shaposhnikov et al. (2014) | 1.260 0.985 1.535 0.27

Vered et al. (2020) | 0.980 0.975 0.986 22.68

Lei et al. (2020) | 1.170 1.026 1.314 0.95

---------------------------+-------------------------------------------------

theta | 1.010 0.995 1.024

-----------------------------------------------------------------------------

Test of theta = 0: z = 137.71 Prob > |z| = 0.0000

Test of homogeneity: Q = chi2(7) = 61.41 Prob > Q = 0.0000

.

**Heterogeneity**

**Publication bias**

meta bias, egger

Effect-size label: RR

Effect size: central

Std. err.: _meta_se

Regression-based Egger test for small-study effects

Random-effects model

Method: Hunter–Schmidt

H0: beta1 = 0; no small-study effects

beta1 = 2.67

SE of beta1 = 0.586

z = 4.55

Prob > |z| = 0.0000

. meta funnelplot

Effect-size label: RR

Effect size: central

Std. err.: _meta_se

Model: Common effect

Method: Inverse-variance

.

**Both-All-AMI**

. meta set central lower upper, civartolerance(1) random(hschmidt) studylabel(authoryear) studysize(samplesize) eslabel(RR)

Meta-analysis setting information

Study information

No. of studies: 3

Study label: authoryear

Study size: samplesize

Effect size

Type: <generic>

Label: RR

Variable: central

Precision

Std. err.: _meta_se

CI: [_meta_cil, _meta_ciu]

CI level: 95%, controlled by level()

User CI: [lower, upper]

User CI level: 95%, controlled by civarlevel()

Model and method

Model: Random effects

Method: Hunter–Schmidt

. meta summarize

Effect-size label: RR

Effect size: central

Std. err.: _meta_se

Study label: authoryear

Meta-analysis summary Number of studies = 3

Random-effects model Heterogeneity:

Method: Hunter–Schmidt tau2 = 0.0000

I2 (%) = 38.15

H2 = 1.62

---------------------------------------------------------------------

Study | RR [95% conf. interval] % weight

-------------------+-------------------------------------------------

Lim et al. (2012) | 1.009 0.995 1.023 42.71

Lee et al. (2010) | 1.068 0.575 1.561 0.07

Lee et al. (2014) | 1.030 1.020 1.040 57.23

-------------------+-------------------------------------------------

theta | 1.021 1.008 1.034

---------------------------------------------------------------------

Test of theta = 0: z = 157.42 Prob > |z| = 0.0000

Test of homogeneity: Q = chi2(2) = 5.76 Prob > Q = 0.0563

.

**Heterogeneity**

**Publication bias**

. meta bias, egger

Effect-size label: RR

Effect size: central

Std. err.: _meta_se

Regression-based Egger test for small-study effects

Random-effects model

Method: Hunter–Schmidt

H0: beta1 = 0; no small-study effects

beta1 = -0.08

SE of beta1 = 1.012

z = -0.08

Prob > |z| = 0.9398

. meta funnelplot

Effect-size label: RR

Effect size: central

Std. err.: _meta_se

Model: Common effect

Method: Inverse-variance

.

**Both genders-Elderly-CVD**

. meta summarize, fixed(invvariance)

Effect-size label: Relative risk

Effect size: RR

Std. err.: _meta_se

Study label: Author

Meta-analysis summary Number of studies = 4

Fixed-effects model Heterogeneity:

Method: Inverse-variance I2 (%) = 33.21

H2 = 1.50

------------------------------------------------------------------------

Study | Relative risk [95% conf. interval] % weight

----------------------+-------------------------------------------------

Zhai et al. (2021) | 1.476 0.222 2.730 0.00

Wang et al. (2013) | 1.008 1.001 1.015 90.47

Ponjoan et al. (2020) | 1.023 1.002 1.044 9.45

Phosri et al. (2020) | 1.198 0.947 1.449 0.07

----------------------+-------------------------------------------------

theta | 1.009 1.003 1.016

------------------------------------------------------------------------

Test of theta = 0: z = 299.21 Prob > |z| = 0.0000

Test of homogeneity: Q = chi2(3) = 4.49 Prob > Q = 0.2130

.

. meta bias, egger fixed

Effect-size label: Relative risk

Effect size: RR

Std. err.: _meta_se

Regression-based Egger test for small-study effects

Fixed-effects model

Method: Inverse-variance

H0: beta1 = 0; no small-study effects

beta1 = 1.29

SE of beta1 = 0.654

z = 1.97

Prob > |z| = 0.0489

**Both genders-Young-CVD**

. meta summarize, random(dlaird)

Effect-size label: Relative risk

Effect size: RR

Std. err.: _meta_se

Study label: Author

Meta-analysis summary Number of studies = 4

Random-effects model Heterogeneity:

Method: DerSimonian–Laird tau2 = 0.0014

I2 (%) = 59.16

H2 = 2.45

------------------------------------------------------------------------

Study | Relative risk [95% conf. interval] % weight

----------------------+-------------------------------------------------

Zhu et al. (2021) | 1.009 1.005 1.013 54.30

Zhai et al. (2021) | 1.171 0.908 1.434 3.82

Ponjoan et al. (2020) | 1.047 0.996 1.098 36.32

Phosri et al. (2020) | 1.221 1.007 1.435 5.56

----------------------+-------------------------------------------------

theta | 1.041 0.988 1.094

------------------------------------------------------------------------

Test of theta = 0: z = 38.32 Prob > |z| = 0.0000

Test of homogeneity: Q = chi2(3) = 7.35 Prob > Q = 0.0616

.

. meta bias, egger random(dlaird)

Effect-size label: Relative risk

Effect size: RR

Std. err.: _meta_se

Regression-based Egger test for small-study effects

Random-effects model

Method: DerSimonian–Laird

H0: beta1 = 0; no small-study effects

beta1 = 1.60

SE of beta1 = 0.602

z = 2.66

Prob > |z| = 0.0078

**Females-All ages -CVD**

. meta summarize, fixed(invvariance)

Effect-size label: Relative risk

Effect size: RR

Std. err.: _meta_se

Study label: Author

Meta-analysis summary Number of studies = 4

Fixed-effects model Heterogeneity:

Method: Inverse-variance I2 (%) = 42.24

H2 = 1.73

------------------------------------------------------------------------

Study | Relative risk [95% conf. interval] % weight

----------------------+-------------------------------------------------

Zhu et al. (2021) | 1.006 1.001 1.011 98.63

Zhai et al. (2021) | 1.537 -0.313 3.387 0.00

Ponjoan et al. (2020) | 1.031 0.990 1.072 1.32

Phosri et al. (2020) | 1.208 0.996 1.420 0.05

----------------------+-------------------------------------------------

theta | 1.006 1.002 1.011

------------------------------------------------------------------------

Test of theta = 0: z = 418.73 Prob > |z| = 0.0000

Test of homogeneity: Q = chi2(3) = 5.19 Prob > Q = 0.1582

.

. meta bias, egger fixed

Effect-size label: Relative risk

Effect size: RR

Std. err.: _meta_se

Regression-based Egger test for small-study effects

Fixed-effects model

Method: Inverse-variance

H0: beta1 = 0; no small-study effects

beta1 = 1.26

SE of beta1 = 0.607

z = 2.07

Prob > |z| = 0.0381

**Males-All ages -CVD**

. meta summarize, fixed(invvariance)

Effect-size label: Relative risk

Effect size: RR

Std. err.: _meta_se

Study label: Author

Meta-analysis summary Number of studies = 4

Fixed-effects model Heterogeneity:

Method: Inverse-variance I2 (%) = 44.87

H2 = 1.81

------------------------------------------------------------------------

Study | Relative risk [95% conf. interval] % weight

----------------------+-------------------------------------------------

Zhu et al. (2021) | 1.017 1.012 1.021 96.80

Zhai et al. (2021) | 1.147 0.899 1.394 0.03

Ponjoan et al. (2020) | 1.032 1.007 1.056 3.12

Phosri et al. (2020) | 1.197 0.991 1.404 0.04

----------------------+-------------------------------------------------

theta | 1.017 1.013 1.022

------------------------------------------------------------------------

Test of theta = 0: z = 460.53 Prob > |z| = 0.0000

Test of homogeneity: Q = chi2(3) = 5.44 Prob > Q = 0.1421

.

. meta bias, egger fixed

Effect-size label: Relative risk

Effect size: RR

Std. err.: _meta_se

Regression-based Egger test for small-study effects

Fixed-effects model

Method: Inverse-variance

H0: beta1 = 0; no small-study effects

beta1 = 1.42

SE of beta1 = 0.625

z = 2.28

Prob > |z| = 0.0226

**Both-Elderly-Stroke**

. meta set central lower upper, civartolerance(1) random(hschmidt) studylabel(authoryear) studysize(samplesize) eslabel(RR)

Meta-analysis setting information

Study information

No. of studies: 4

Study label: authoryear

Study size: samplesize

Effect size

Type: <generic>

Label: RR

Variable: central

Precision

Std. err.: _meta_se

CI: [_meta_cil, _meta_ciu]

CI level: 95%, controlled by level()

User CI: [lower, upper]

User CI level: 95%, controlled by civarlevel()

Model and method

Model: Random effects

Method: Hunter–Schmidt

. meta summarize

Effect-size label: RR

Effect size: central

Std. err.: _meta_se

Study label: authoryear

Meta-analysis summary Number of studies = 4

Random-effects model Heterogeneity:

Method: Hunter–Schmidt tau2 = 0.0008

I2 (%) = 86.09

H2 = 7.19

-------------------------------------------------------------------------

Study | RR [95% conf. interval] % weight

-----------------------+-------------------------------------------------

Ponjoan et al. (2020) | 1.032 0.990 1.074 19.18

Lim et al. (2017) | 1.027 1.007 1.046 26.89

Lichtman et al. (2016) | 1.091 1.072 1.109 27.19

He et al. (2021) | 1.026 1.006 1.046 26.74

-----------------------+-------------------------------------------------

theta | 1.045 1.015 1.076

-------------------------------------------------------------------------

Test of theta = 0: z = 67.32 Prob > |z| = 0.0000

Test of homogeneity: Q = chi2(3) = 30.40 Prob > Q = 0.0000

. meta forestplot

Effect-size label: RR

Effect size: central

Std. err.: _meta_se

Study label: authoryear

**Heterogeneity**

**Publication bias**

. meta bias, egger

Effect-size label: RR

Effect size: central

Std. err.: _meta_se

Regression-based Egger test for small-study effects

Random-effects model

Method: Hunter–Schmidt

H0: beta1 = 0; no small-study effects

beta1 = -1.76

SE of beta1 = 3.319

z = -0.53

Prob > |z| = 0.5960

. meta funnelplot

Effect-size label: RR

Effect size: central

Std. err.: _meta_se

Model: Common effect

Method: Inverse-variance

.

**Both-Young-Stroke**

. meta set central lower upper, civartolerance(1) random(hschmidt) studylabel(authoryear) studysize(samplesize) eslabel(RR)

Meta-analysis setting information

Study information

No. of studies: 5

Study label: authoryear

Study size: samplesize

Effect size

Type: <generic>

Label: RR

Variable: central

Precision

Std. err.: _meta_se

CI: [_meta_cil, _meta_ciu]

CI level: 95%, controlled by level()

User CI: [lower, upper]

User CI level: 95%, controlled by civarlevel()

Model and method

Model: Random effects

Method: Hunter–Schmidt

. meta summarize

Effect-size label: RR

Effect size: central

Std. err.: _meta_se

Study label: authoryear

Meta-analysis summary Number of studies = 5

Random-effects model Heterogeneity:

Method: Hunter–Schmidt tau2 = 0.0033

I2 (%) = 92.15

H2 = 12.75

-------------------------------------------------------------------------

Study | RR [95% conf. interval] % weight

-----------------------+-------------------------------------------------

Zhu et al. (2021) | 1.013 0.994 1.033 21.75

Ponjoan et al. (2020) | 0.978 0.914 1.041 17.04

Lim et al. (2017) | 0.995 0.958 1.032 20.19

Lichtman et al. (2016) | 1.156 1.126 1.186 20.93

He et al. (2021) | 1.040 1.001 1.078 20.08

-----------------------+-------------------------------------------------

theta | 1.039 0.985 1.092

-------------------------------------------------------------------------

Test of theta = 0: z = 37.99 Prob > |z| = 0.0000

Test of homogeneity: Q = chi2(4) = 73.78 Prob > Q = 0.0000

.

**Heterogeneity**

**Publication bias**

. meta bias, egger

Effect-size label: RR

Effect size: central

Std. err.: _meta_se

Regression-based Egger test for small-study effects

Random-effects model

Method: Hunter–Schmidt

H0: beta1 = 0; no small-study effects

beta1 = -3.52

SE of beta1 = 3.822

z = -0.92

Prob > |z| = 0.3572

. meta funnelplot

Effect-size label: RR

Effect size: central

Std. err.: _meta_se

Model: Common effect

Method: Inverse-variance

.

**Female-All-Stroke**

. meta set central lower upper, civartolerance(1) random(hschmidt) studylabel(authoryear) studysize(samplesize) eslabel(RR)

Meta-analysis setting information

Study information

No. of studies: 5

Study label: authoryear

Study size: samplesize

Effect size

Type: <generic>

Label: RR

Variable: central

Precision

Std. err.: _meta_se

CI: [_meta_cil, _meta_ciu]

CI level: 95%, controlled by level()

User CI: [lower, upper]

User CI level: 95%, controlled by civarlevel()

Model and method

Model: Random effects

Method: Hunter–Schmidt

. meta summarize

Effect-size label: RR

Effect size: central

Std. err.: _meta_se

Study label: authoryear

Meta-analysis summary Number of studies = 5

Random-effects model Heterogeneity:

Method: Hunter–Schmidt tau2 = 0.0002

I2 (%) = 65.94

H2 = 2.94

------------------------------------------------------------------------

Study | RR [95% conf. interval] % weight

----------------------+-------------------------------------------------

Zhu et al. (2021) | 1.003 0.983 1.022 24.98

Ponjoan et al. (2020) | 1.087 1.005 1.169 4.26

Lim et al. (2017) | 1.022 0.990 1.054 16.95

Vered et al. (2020) | 0.980 0.972 0.988 33.49

He et al. (2021) | 1.027 1.000 1.053 20.31

----------------------+-------------------------------------------------

theta | 1.007 0.989 1.025

------------------------------------------------------------------------

Test of theta = 0: z = 108.72 Prob > |z| = 0.0000

Test of homogeneity: Q = chi2(4) = 24.01 Prob > Q = 0.0001

.

**Heterogeneity**

**Publication bias**

. meta bias, egger

Effect-size label: RR

Effect size: central

Std. err.: _meta_se

Regression-based Egger test for small-study effects

Random-effects model

Method: Hunter–Schmidt

H0: beta1 = 0; no small-study effects

beta1 = 3.52

SE of beta1 = 0.740

z = 4.76

Prob > |z| = 0.0000

. meta funnelplot

Effect-size label: RR

Effect size: central

Std. err.: _meta_se

Model: Common effect

Method: Inverse-variance

.

**Male-All-Stroke**

. meta set central lower upper, civartolerance(1) random(hschmidt) studylabel(authoryear) studysize(samplesize) eslabel(RR)

Meta-analysis setting information

Study information

No. of studies: 5

Study label: authoryear

Study size: samplesize

Effect size

Type: <generic>

Label: RR

Variable: central

Precision

Std. err.: _meta_se

CI: [_meta_cil, _meta_ciu]

CI level: 95%, controlled by level()

User CI: [lower, upper]

User CI level: 95%, controlled by civarlevel()

Model and method

Model: Random effects

Method: Hunter–Schmidt

. meta summarize

Effect-size label: RR

Effect size: central

Std. err.: _meta_se

Study label: authoryear

Meta-analysis summary Number of studies = 5

Random-effects model Heterogeneity:

Method: Hunter–Schmidt tau2 = 0.0003

I2 (%) = 79.99

H2 = 5.00

------------------------------------------------------------------------

Study | RR [95% conf. interval] % weight

----------------------+-------------------------------------------------

Zhu et al. (2021) | 1.028 1.011 1.046 23.85

Ponjoan et al. (2020) | 1.044 0.969 1.119 5.63

Lim et al. (2017) | 1.024 1.004 1.045 22.23

Vered et al. (2020) | 0.985 0.977 0.993 27.86

He et al. (2021) | 1.030 1.006 1.054 20.43

----------------------+-------------------------------------------------

theta | 1.017 0.997 1.036

------------------------------------------------------------------------

Test of theta = 0: z = 100.58 Prob > |z| = 0.0000

Test of homogeneity: Q = chi2(4) = 36.25 Prob > Q = 0.0000

.

**Heterogeneity**

**Publication bias**

. meta bias, egger

Effect-size label: RR

Effect size: central

Std. err.: _meta_se

Regression-based Egger test for small-study effects

Random-effects model

Method: Hunter–Schmidt

H0: beta1 = 0; no small-study effects

beta1 = 2.71

SE of beta1 = 1.075

z = 2.52

Prob > |z| = 0.0117

. meta funnelplot

Effect-size label: RR

Effect size: central

Std. err.: _meta_se

Model: Common effect

Method: Inverse-variance

.
